# Mutations in the B30.2 and the central helical scaffold domains of pyrin differentially affect inflammasome activation

**DOI:** 10.1038/s41419-023-05745-9

**Published:** 2023-03-25

**Authors:** Daria Chirita, Pauline Bronnec, Flora Magnotti, Sarah Dalmon, Amandine Martin, Michel Popoff, Mathieu Gerfaud-Valentin, Pascal Sève, Alexandre Belot, Anne Contis, Agnes Duquesne, Gaetane Nocturne, Irene Lemelle, Sophie Georgin-Lavialle, Guilaine Boursier, Isabelle Touitou, Yvan Jamilloux, Thomas Henry

**Affiliations:** 1 CIRI, Centre International de Recherche en Infectiologie, Univ Lyon, Inserm U1111, Université Claude Bernard Lyon 1, CNRS, UMR5308, ENS de Lyon, F-69007 LYON, France; 2 Bacterial Toxins, Institut Pasteur, Paris, France; 3 Department of Internal Medicine, University Hospital Croix-Rousse, Lyon 1 University, Lyon, France; 4 LIFE, Lyon Immunopathology FEderation, Lyon, France; 5 Department of Pediatric Nephrology, Rheumatology, Dermatology, Reference centre for Rheumatic, AutoImmune and Systemic diseases in children (RAISE), Hôpital Femme Mère Enfant, CHU Lyon, Bron, France; 6 Department of Internal Medicine, Saint André Hospital, CHU Bordeaux, Bordeaux, France; 7 Department of Rheumatology, Université Paris-Saclay, INSERM UMR1184: Center for immunology of viral infections and autoimmune diseases, Assistance Publique-Hôpitaux de Paris (AP-HP), Hôpital Bicêtre, Le Kremlin Bicêtre, France; 8 Paediatric onco-haematology, University Hospital of Nancy – Children’s hospital, Vandoeuvre-Lès-Nancy, France; 9 Sorbonne University, department of internal medicine, Tenon hospital, DMU 3ID, AP-HP, National reference center for autoinflammatory diseases and inflammatory Amyloidosis (CeRéMAIA), INSERM U938, Paris, France; 10 Department of Molecular genetics and Cytogenomics, CHU Montpellier, Univ Montpellier, Reference Center for Autoinflammatory Diseases and Amyloidosis (CeRéMAIA), Montpellier, France

**Keywords:** Cell death and immune response, Immunological disorders

## Abstract

Familial Mediterranean Fever (FMF) is the most common monogenic autoinflammatory disorder. FMF is caused by mutations in the *MEFV* gene, encoding pyrin, an inflammasome sensor. The best characterized pathogenic mutations associated with FMF cluster in exon 10. Yet, mutations have been described along the whole *MEFV* coding sequence. Exon 10 encodes the B30.2 domain of the pyrin protein, but the function of this human-specific domain remains unclear. Pyrin is an inflammasome sensor detecting RhoA GTPase inhibition following exposure to bacterial toxins such as TcdA. Here, we demonstrate that the B30.2 domain is dispensable for pyrin inflammasome activation in response to this toxin. Deletion of the B30.2 domain mimics the most typical FMF-associated mutation and confers spontaneous inflammasome activation in response to pyrin dephosphorylation. Our results indicate that the B30.2 domain is a negative regulator of the pyrin inflammasome that acts independently from and downstream of pyrin dephosphorylation. In addition, we identify the central helical scaffold (CHS) domain of pyrin, which lies immediately upstream of the B30.2 domain as a second regulatory domain. Mutations affecting the CHS domain mimic pathogenic mutations in the B30.2 domain and render the pyrin inflammasome activation under the sole control of the dephosphorylation. In addition, specific mutations in the CHS domain strongly increase the cell susceptibility to steroid catabolites, recently described to activate pyrin, in both a cell line model and in monocytes from genotype-selected FMF patients. Taken together, our work reveals the existence of two distinct regulatory regions at the C-terminus of the pyrin protein, that act in a distinct manner to regulate positively or negatively inflammasome activation. Furthermore, our results indicate that different mutations in pyrin regulatory domains have different functional impacts on the pyrin inflammasome which could contribute to the diversity of pyrin-associated autoinflammatory diseases.

## Introduction

Inflammasomes are multiprotein platforms assembled in the cytosol upon sensing of microbial infections, danger signals, or alteration of the cell homeostasis [1]. Inflammasome assembly leads to activation of the pro-inflammatory caspase-1, release of IL-1β and IL-18. In most cases, inflammasome activation leads to a fast cell death, termed pyroptosis which results from caspase-1-mediated cleavage of the pore-forming protein, gasdermin D. Inflammasomes participate in the innate immune defenses [2]. Inflammasomes are also involved in deleterious inflammatory processes. Particularly, mutations in genes encoding various inflammasome sensors are responsible for rare autoinflammatory diseases [3].

Pyrin is an inflammasome sensor acting as a guard of RhoA GTPases activity. Various bacterial toxins (such as *Clostridioides difficile* toxins A/B, TcdA/B) or effectors inhibit RhoA and trigger pyrin activation. The mechanisms linking RhoA inhibition to pyrin activation have been partly solved [4–6]. At steady state, RhoA activates its effectors including PKN1 and PKN2, two kinases that phosphorylate pyrin on two serine residues (S208 and S242). Phosphorylated pyrin interacts with 14-3-3 chaperone proteins that sequester pyrin away from the inflammasome adaptor, ASC [7–9]. Inhibition of RhoA and the ensuing deactivation of PKN1/2 leads to the PP2A-mediated dephosphorylation of pyrin [10]. Yet, this is not sufficient to trigger pyrin inflammasome activation. Another poorly understood mechanism controls pyrin inflammasome activation downstream of pyrin dephosphorylation in a microtubule-dependent manner [4, 7, 9]. In addition to RhoA inhibition, we and others recently identified steroid metabolites/analogues that can activate pyrin in a B30.2-dependent manner [11, 12].

Mutations in *MEFV*, the gene encoding pyrin, cause two autoinflammatory diseases, familial Mediterranean fever (FMF) and pyrin-associated autoinflammation with neutrophilic dermatosis (PAAND) [5, 13–15]. PAAND are caused by mutations affecting the phosphorylated serine or neighboring residues involved in the interaction with 14-3-3 proteins [5, 16]. The link between genotype and phenotype in FMF is more complex and less understood [17]. The prototypical FMF disease is a recessive disease due to bi-allelic mutations in *MEFV* exon 10, encoding pyrin B30.2 domain with the most frequent pathogenic mutation being p.M694V. FMF-associated *MEFV* mutations deregulate the pyrin inflammasome in monocytes by lifting the requirement for an intact microtubule network [9, 18], by invalidating pyrin regulatory mechanism 2 and leaving its activation under the sole control of its dephosphorylation [4]. These mutations result in pyrin inflammasome demonstrating a lower threshold of activation to specific pyrin agonists [19] and higher responses to LPS exposure [20]. Beside *MEFV* exon 10 mutations, a number of patients with a clinical diagnosis of FMF have *MEFV* mutations affecting other exons [17]. In addition, a number of these mutations results in FMF diseases with a dominant inheritance pattern [21, 22]. Most of these mutations are of unknown significance due to their rare frequency in the human population and due to lack of knowledge on the role of affected domain in pyrin regulation.

Here, we studied the role of the two C-terminal domains of pyrin in inflammasome activation in response to diverse stimuli to increase our knowledge on the regulatory mechanisms controlling pyrin inflammasome activation and the impact of FMF-associated mutations.

## Results

### B30.2 domain is dispensable for pyrin activation in response to TcdA

**Fig. 1:**
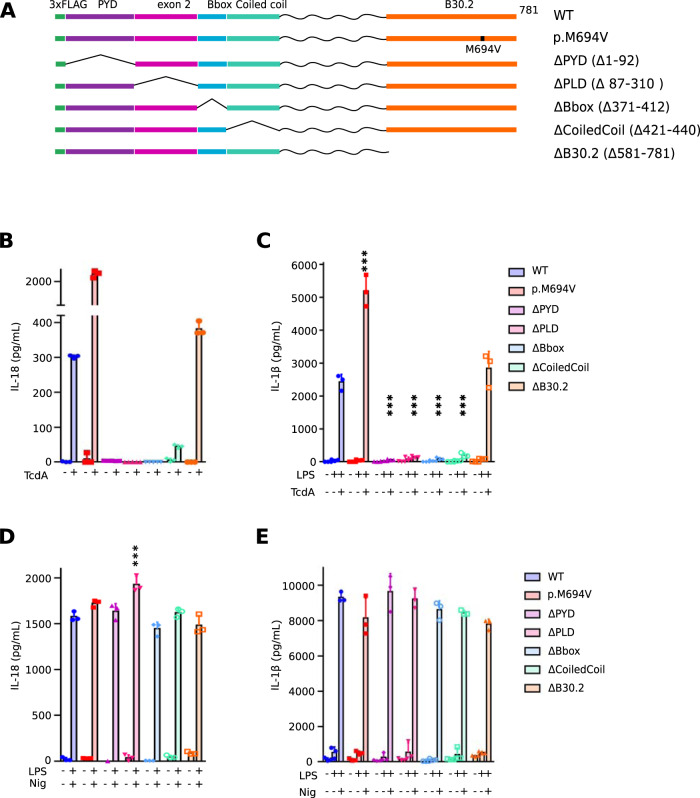
B30.2 is dispensable for TcdA-mediated pyrin activation. **A** Schematic representation of pyrin variants. U937 monocytes (**B**, **D**) or macrophages (**C**, **E**) incubated with doxycycline to induce expression of the indicated pyrin variants, were treated with TcdA for 6 h (**B**, **C**) or with LPS for 3 h followed by Nigericin for 3 h (**D**, **E**). The concentration of IL-18 (**B**, **D**) and IL-1β (**C**, **E**) in the supernatant was determined by ELISA. Data information: Data from one experiment representative of three independent experiments. Mean, SD of triplicates and individual data points are shown. **C**–**E** Ordinary one-way ANOVA and Dunnett’s multiple comparisons tests were performed to compare U937 cells expressing WT pyrin to the other cell lines. **C** ***: *p* < 0.001, (**D**) ***: *p* < 0.001.

Pyrin is a multidomain protein containing a PYD domain, a linker domain containing the two critical phosphorylation residues and encoded by exon 2 (phosphorylated linker domain-PLD), a B-box domain, a central helical scaffold and a B30.2 domain. The role of each domain in pyrin inflammasome activation is still unclear. To answer this question, we used the human monocytic cell line, U937 and generated cell lines stably expressing doxycycline-inducible pyrin variants that lack one of the domains (Fig. 1A). U937 cells stably expressing the classical FMF-associated *MEFV* variant p.M694V were included as a control. The obtained monocytes were first stimulated with *C. difficile* toxin A (TcdA) and IL-18 release was monitored as an inflammasome readout (Fig. 1B). Cells expressing the pyrin variant lacking the B30.2 domain (ΔB30.2) responded to TcdA and secreted similar or even slightly higher IL-18 levels than cells expressing WT pyrin. In contrast, all the other domain deletions strongly decreased (coiled-coil domain deletion) or abolished IL-18 secretion in response to TcdA. Cells expressing the p.M694V pyrin variant secreted higher level of IL-18 than cells expressing WT pyrin in agreement with the hyper-reactivity of primary monocytes from FMF patients to clostridial toxins [19, 23]. Undifferentiated U937 cells are deficient for IL-1β production. U937 cells were thus differentiated into macrophages using PMA, and LPS was used to induce proIL-1β expression. PMA-differentiated U937 macrophages expressing ΔB30.2 pyrin variant released high levels of IL-1β (Fig. 1C) while all the other cell lines expressing deletion mutants led to minimal IL-1β secretion. No major differences in IL-18 or IL-1β secretion were observed following treatment with the NLRP3-specific stimulus LPS + nigericin (Fig. 1D, E). In the absence of doxycycline (i.e., of pyrin variants induction), TcdA did not trigger IL-1β or IL-18 release (Supplemental Fig. S1). These results show that the pyrin PLD, the B-box and the coiled-coil domains are specifically required for inflammasome cytokines secretion in response to the pyrin-specific stimulus TcdA. Furthermore, these results indicated that the B30.2 domain is dispensable for pyrin activation in response to TcdA.

### Deletion of the B30.2 domain suppresses pyrin regulatory step 2

**Fig. 2:**
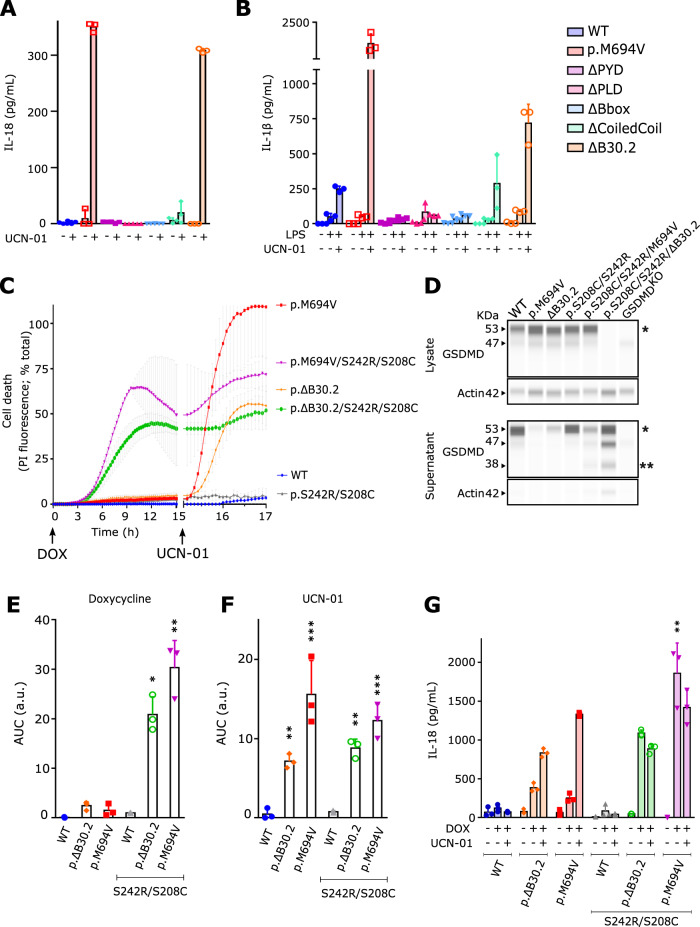
Deletion of the B30.2 domain suppresses pyrin regulation step 2. **A**, **B** U937 monocytes (**A**) or macrophages (**B**) incubated with doxycycline to induce pyrin expression were treated with UCN-01 for 3 h. IL-18 (**A**) and IL-1β (**B**) concentration were determined in the supernatant by ELISA. **C** Cell death kinetics upon doxycycline addition followed by UCN-01 treatment were determined in U937 monocytes expressing the indicated pyrin variants. PI incorporation/fluorescence was monitored every 15 min for the indicated time. **D** U937 monocytes were incubated with doxycycline for 9 h, GSDMD cleavage was analyzed in the cell lysate (top panels) and the cell supernatant (bottom panels) by capillary-based Western blot analysis. * and ** indicate the full length and the cleaved GSDMD bands, respectively. **E**, **F** Areas Under the Curve (AUC) corresponding to the above kinetics are shown. **G** U937 monocytes were treated with doxycycline for 16 h followed by UCN-01 addition for 2 h, IL-18 concentration in the supernatant was measured by ELISA. Data information: Data from one experiment representative of one (**D**) to three (**A**–**C**; **E**–**G**) independent experiments. Mean, SD of triplicates and individual data points are shown. **E** a.u.: arbitrary units. **A**, **B** Kruskal–Wallis and Dunn’s multiple comparisons tests and (**F**, **G**) ordinary one-way ANOVA and Dunnett’s multiple comparisons tests were performed to compare U937 cells expressing WT pyrin to the other cell lines. **E** *: *p* = 0.0232; **: *p* = 0.0038; (**F**) ** (left to right): *p* = 0.0076, 0.0014; ***: *p* < 0.001; (**G**) ***: *p* < 0.001.

We have previously demonstrated that FMF-associated p.M694V/I or p.M680I mutations lead to the loss of a regulatory mechanism and to the exclusive control of the pyrin inflammasome by phosphorylation/dephosphorylation [4]. This defect can be functionally evidenced by treating cells with a PKC superfamily inhibitor UCN-01, that inhibits PKN1 and PKN2, leading to pyrin dephosphorylation [4]. Interestingly, ΔB30.2 pyrin expressing cells released high levels of IL-18 in response to UCN-01. As expected, cells expressing WT pyrin did not release IL-18 in contrast to cells expressing p.M694V variant (Fig. 2A). Similarly, upon differentiation into macrophages and treatment with UCN-01, only cells expressing p.M694V or ΔB30.2 *MEFV* variants led to a greater secretion of IL-1β than WT pyrin-expressing cells (Fig. 2B). In the absence of doxycycline, UCN-01 did not trigger IL-1β or IL-18 release (Supplemental Fig. S2A, B). These results suggest that deletion of the B30.2 domain abolishes the second pyrin regulatory mechanism to leave pyrin activation under the sole control of pyrin dephosphorylation.

To confirm this phenotype genetically without relying on a chemical inhibitor, we generated a U937 cell line invalidated for step 1 inhibitory mechanism (p.S08C/S242R phospho-null mutations) on a ΔB30.2 background. The phospho-null mutations combined with the p.M694V mutation (p.S208C/S242R/M694V) yield to a constitutively activated pyrin inflammasome [4]. The corresponding cell line was thus used as a positive control. Importantly, doxycycline-mediated induction of p.S208C/S242R/ΔB30.2 pyrin variant was sufficient to trigger cell death (Fig. 2C, E) and to promote GSDMD cleavage as suggested by the pattern of GSDMD immunodetection in the cell supernatant (Fig. 2D). The cell death was significantly blocked by the caspase-1 inhibitor, VX-765 (Supplemental Fig. S2C, D). Altogether, these results demonstrate the p.S208C/S242R/ΔB30.2 pyrin variant is constitutively activated and triggers pyroptosis upon expression. Due to the high doxycycline-induced cell death, these cell lines barely responded to UCN-01 stimulation in contrast to p.M694V or ΔB30.2 single mutants (Fig. 2C, F). Similarly, induction of the p.S208C/S242R/ΔB30.2 pyrin protein led to potent IL-18 secretion in the absence of any additional stimulus (Fig. 2G). These results indicate that deletion of the B30.2 domain fully invalidated pyrin regulatory mechanism 2 and, when combined with mutations invalidating pyrin regulatory mechanism 1, led to constitutive activation of this pyrin variant and spontaneous pyroptosis. Taken together these results demonstrate that the B30.2 domain of pyrin acts as a negative regulator of pyrin activation following TcdA treatment, UCN-01-mediated dephosphorylation or genetic invalidation of phosphorylation sites.

### The B30.2 domain-mediated regulation acts downstream of pyrin dephosphorylation, upstream of ASC speck formation and, independently from caspase-1

**Fig. 3:**
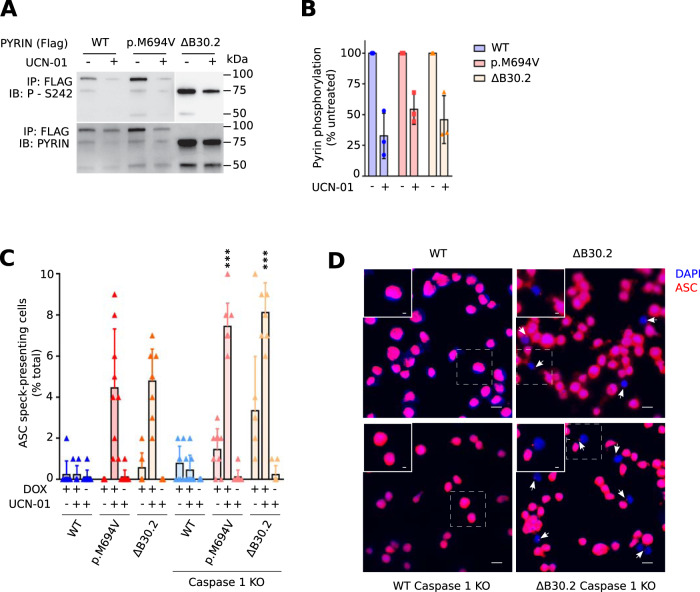
The B30.2-mediated regulation acts downstream of dephosphorylation, and independently of Caspase-1. **A**, **B** Doxycycline-induced U937 monocytes expressing pyrin WT, p.M694V or pyrin deleted from the B30.2 domain were treated with UCN-01 for 15 min. **A** Pyrin phosphorylation at the S242 site was assessed by Western blot following immunoprecipitation and (**B**) quantified by densitometry. **C**, **D** U937 monocytes WT or *Caspase-1* knock-out (KO) were treated with doxycycline for 15 h and/or with UCN-01 for 25 min. ASC speck formation (indicated with white arrows) was monitored by immunofluorescence. Quantification (**C**) and representative images (**D**) are shown. Data information: (**A**, **C**) Data from one experiment representative of three independent experiments. **B** Values from individual experiments, mean and SD from three independent experiments are presented. Pyrin S242 phosphorylation was quantified using ImageJ software and reported as percentage of the untreated condition of the corresponding cell line. Each point represents the densitometry quantification from one experiment. **C** Values from individual experiments, mean and SD from three independent experiments are presented. Each dot corresponds to the percentage of ASC specks found in approximately 100 cells counted per condition. Kruskal–Wallis and Dunn’s multiple comparisons tests were performed. * (left to right): *p* = 0.0306, 0.0351, 0.0494; ***: *p* < 0.001. **D** Scale bars: 10 µm and 2,5 µm in the main figures and insets, respectively.

The above results suggest that pyrin B30.2 domain-mediated regulation acts on pyrin regulatory mechanism 2 or downstream and not on the first regulatory mechanism associated with pyrin phosphorylation/dephosphorylation. Since the impact of FMF-associated, B30.2-affecting point mutations on phosphorylation is unclear [4, 6, 7, 9, 24], we investigated the impact of B30.2 deletion on pyrin dephosphorylation. Although at steady state, the ΔB30.2 pyrin protein was expressed at higher level than WT or p.M694V protein variants (possibly reflecting a greater stability), we could not detect any impact of the B30.2 deletion on UCN-01-mediated pyrin dephosphorylation (Fig. 3A, B). The B30.2-mediated regulation thus likely occurs downstream of pyrin dephosphorylation.

To further map the B30.2-mediated regulation along the pyrin inflammasome activation process, we monitored ASC speck formation following UCN-01 treatment. As expected, and in line with the results presented above, UCN-01-mediated pyrin dephosphorylation was sufficient to trigger ASC speck formation in cells expressing p.M694V or ΔB30.2 pyrin variants (Fig. 3C, D). Cells expressing WT pyrin did not display any increase in ASC speck formation in the presence of UCN-01 (i.e., upon pyrin dephosphorylation). These results thus indicate that the B30.2-mediated regulation takes place downstream of pyrin dephosphorylation but upstream of ASC speck formation.

The B30.2 domain has been shown to interact with caspase-1 leading to the hypothesis that the B30.2 domain could regulate pyrin inflammasome through caspase-1 inhibition [25–27]. Yet, the above results demonstrate that the B30.2 domain regulates pyrin activation upstream of ASC speck formation suggesting that it may act independently of caspase-1. To validate this finding, we transduced the various pyrin mutants in caspase-1-deficient cells (Supplemental Fig. S3) and quantified ASC speck formation in response to UCN-01 (Fig. 3C, D). ASC speck formation was slightly higher in these cells likely due to the lack of caspase-1-mediated pyroptosis. Yet, in the absence of caspase-1, UCN-01 increased ASC speck formation in ΔB30.2 pyrin-expressing cells but not in WT pyrin-expressing cells clearly indicating that B30.2 negatively regulates pyrin inflammasome activation downstream of the dephosphorylation, upstream of ASC speck formation and independently of caspase-1. Since p.M694V mimics B30.2 deletion for all the above assays, these results suggest that the p.M694V mutation fully abolishes the negative regulatory function of this domain. This exon 10-encoded mutation may thus be classified as a loss-of-function mutation with regard to the functionality of the B30.2 domain (loss of the negative regulation) and thus a as gain of function in term of the full protein.

### Mutations in the central helical scaffold phenocopy B30.2 pathogenic mutations in terms of UCN-01-mediated responses

Structural data on the C-terminal part of the pyrin protein indicates that the B30.2 domain interacts with another domain, termed the central helical scaffold which is made of coiled-coil α helices. This 272 amino acid-long domain (from 414 to 586), encoded by *MEFV* exons 3–8, has a function largely unknown and mutations affecting this domain mostly result in *MEFV* variants of unknown significance [28].

**Fig. 4:**
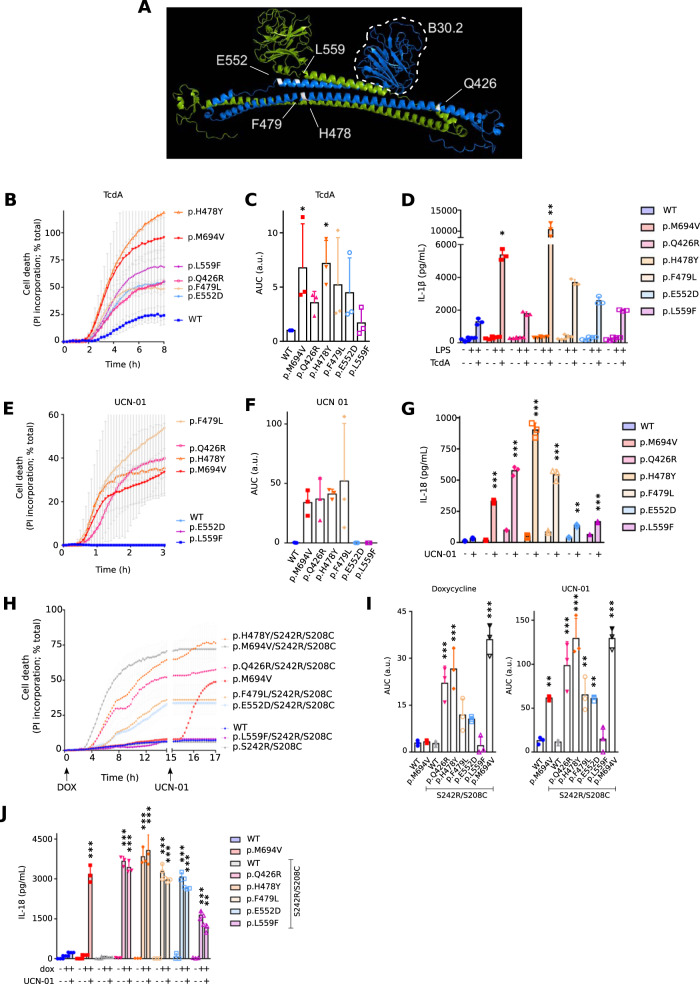
Mutations in the Central Helical Scaffold (CHS) domain phenocopy the classical FMF-associated B30.2 mutation - p.M694V - in terms of UCN-01 and TcdA-mediated responses. **A** CHS mutation sites (white) within the known crystal structure of the CHS and the B30.2 domains. **B**, **C**, **E**–**G** U937 monocytes or (**D**) macrophages incubated with doxycycline to induce expression of the indicated variants, were treated with (**B**–**D**) TcdA for 8 h, or (**E**–**G**) UCN-01 for 3 h. **B**, **E** Cell death kinetics was monitored through PI incorporation/fluorescence every 5 min over the indicated time. **C**, **F** Areas Under the Curve (AUC) corresponding to the kinetics are shown. **D** IL-1β or (**G**) IL-18 concentration in the supernatant was determined by ELISA. **H**–**J** U937 monocytes were treated with doxycycline for 16 h followed by UCN-01 for 2 h. **H** PI incorporation/fluorescence was monitored every 15 min for the indicated time, (**I**) corresponding AUC are shown. **J** IL-18 concentration in the supernatant was determined by ELISA. Data information: (**B**, **C**–**J**) Data from one experiment representative of three independent experiments, (**C**) AUC from three independent experiments are shown. Mean and SD of triplicates and individual data points are shown. **B**, **G** a.u.: arbitrary units. **B**,**D** Kruskal–Wallis and Dunn’s multiple comparisons tests or (**E**, **H**, **J**, **K**) ordinary one-way ANOVA and Dunnett’s multiple comparisons tests were performed to compare U937 cells expressing WT pyrin to those expressing other variants. **(C**) * (left to right): *p* = 0.0415, 0.0277; (**D**) *: *p* = 0.0184; **: *p* = 0.0023; (**E**) ***: *p* < 0.001; (**G**) **: *p* = 0.0054; ***: *p* < 0.001; (**I**) ** (left to right): *p* = 0.0055, 0.0029, 0.0061; ***: *p* < 0.001; (**J**) **: *p* = 0.0026; ***: *p* < 0.001.

We thus generated five distinct U937 cell lines expressing *MEFV* variants of unknown significance identified in patients [21, 28, 29]. These variants (p.Q426R; p.H478Y; p.F479L; p.E552D; p.L559F) present mutations in various section of the CHS domain (Fig. 4A). All these constructs were expressed under a doxycycline-inducible promoter. Addition of doxycycline induced all these variants (Supplemental Fig. S4A) but did not trigger substantial cell death indicating that the corresponding proteins are not constitutively activated. We then assessed the impact of each of this variant on TcdA-triggered responses. Cells expressing the dominant p.H478Y variant behaved like cells expressing p.M694V variant and underwent cell death significantly faster than WT *MEFV*-expressing cells (Fig. 4B, C). A similar trend, although not statistically different from WT was observed for cells expressing p.Q426R, p.F479L, or p.L559F variants. Cells expressing p.E552D *MEFV* variant had a milder and more variable phenotype than all the other cell lines. LPS + nigericin-mediated cell death was similar in all the cell lines (with some kinetics differences potentially linked to a cross-talk between pyrin and NLRP3 inflammasomes [20]) (Supplemental Fig. S4B–D). IL-1β quantification led to the same conclusions with TcdA-mediated responses being statistically greater in cells expressing p.H478Y and p.M694V *MEFV* variants (Fig. 4D). Thus, in agreement with Honda and colleagues [23], we observed that different mutations within *MEFV*, including p.M694V, lead to different level of enhancement of TcdA-triggered responses. This suggests that different CHS-targeting mutations may cause different level of deregulation of the pyrin inflammasome.

**Fig. 5:**
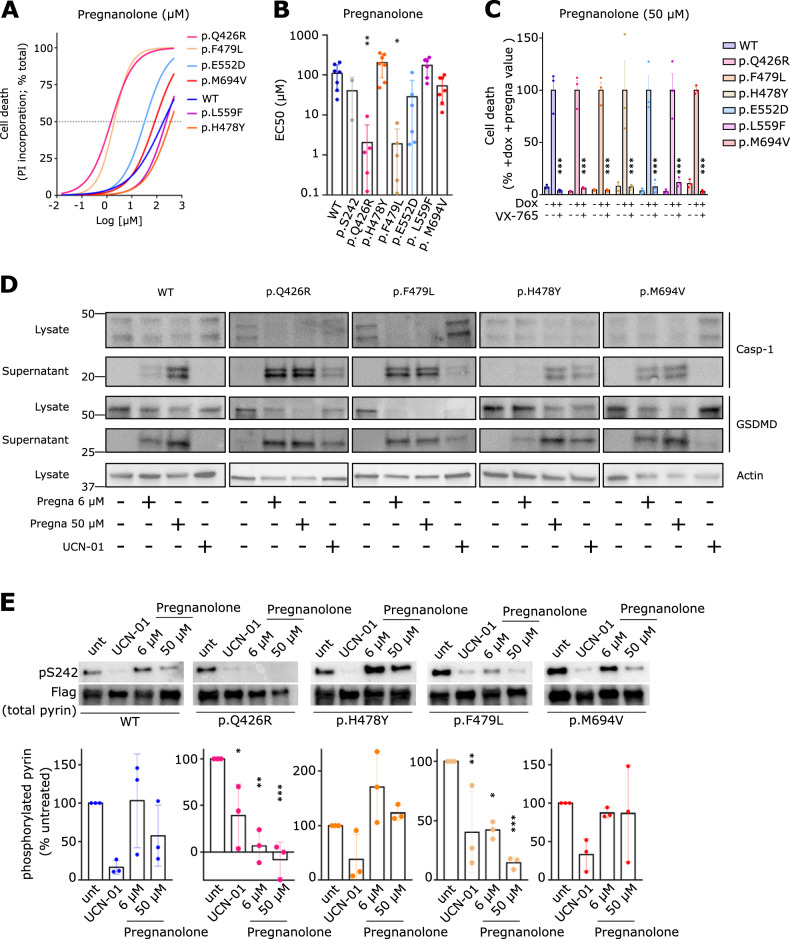
Specific mutations in the CHS domain render pyrin highly sensitive to steroid catabolites. U937 monocytes incubated with doxycycline (unless otherwise indicated (**C**)) to induce expression of the indicated pyrin variants, were treated (**A**, **B**) with decreasing doses of pregnanolone or (**C**–**E**) the indicated stimuli. **A** Cell death levels were measured at 3 h post-treatment in response to varying concentrations of pregnanolone. **B** EC50 was determined at 3 h post-treatment. **C** Cell death levels were measured at 3 h post-treatment in the absence of doxycycline or in the presence of VX-765. Results were normalized to the cell death value of cells treated with doxycycline and pregnanolone at 50 μM. **D** Caspase-1 (top panels) and GSDMD (bottom panels) cleavage were monitored in the cell lysate or the cell supernatant at 90 min post-UCN-01 addition or at 3 h post pregnanolone addition. **E** Cells were treated with UCN-01 for 15 min or with VX765 for 30 min followed by pregnanolone at the indicated dose for 30 min. Pyrin phosphorylation at the S242 site was assessed by Western blot following immunoprecipitation and quantified by densitometry. Data information: (**A**) Data from one experiment representative of three independent experiments. The curve was obtained by an ordinary (Least squares) fit using the log (agonist) vs. normalized response-variable slope model. **B** EC values were obtained from the above analysis using best-fit values. Each point represents the mean EC50 calculated from one biological triplicate. The bars represent the mean and SD of 3–7 independent experiments. Kruskal–Wallis and Dunn’s multiple comparisons tests for the panel on the left were performed. **C** Data from one experiment. Each point represents the value of one well, mean and SEM are shown. **D** Data from one experiment. (E-Top panel) Images from one experiment representative of three independent experiments are shown. (Bottom panel): Each point represents the densitometry quantification (normalized to the untreated sample) from one experiment. The bars represent the mean and SD of 3 independent experiments. **B** Ordinary one-way ANOVA and Dunnett’s multiple comparisons tests were performed. **: *p* = 0.0078; *: *p* = 0.0168. **C** One way Anova with Sidak’s correction for multiple test was applied. Two-tailed *p* values are shown. ***: *p* < 0.001. **E** * (left to right): *p* = 0.0198, 0.0104; ** (left to right): *p* = 0.0017, 0.0088; ***: *p* < 0.001.

To confirm this, we switched to UCN-01, which better discriminates whether the variants behave like the prototypical p.M694V variant [4, 23, 30]. Three of these variants-expressing cell lines (p.Q426R, p.H478Y and p.F479L) responded to UCN-01 and underwent a rapid cell death, previously characterized as a pyroptotic cell death (Fig. 4E, F) and associated with the appearance of cleaved Caspase-1 and cleaved GSDMD in the cell supernatant (Fig. 5D). This inflammasome response was confirmed by quantifying IL-18 secretion (Fig. 4G). To genetically strengthen these findings, the 5 CHS mutations were combined to p.S208C/p.S242R mutations. These results (Fig. 4H–J) confirmed the major phenotype of p.Q426R, p.H478Y (and as expected p.M694V). p.F479L, also caused substantial deregulation of the pyrin inflammasome responses when combined to p.S208C/p.S242R mutations although the difference with WT pyrin was not statistically significant in all the assays.

Overall, these results indicate that specific CHS-affecting mutations mimic pathogenic FMF mutations affecting the B30.2 domain (e.g., p.M694V) and activate the pyrin inflammasome following dephosphorylation. Based on these cellular phenotypes, they should thus be qualified as pathogenic mutations. Interestingly, these assays clearly indicate that the different mutations have different quantitative impact on the pyrin inflammasome mirroring the clinical FMF presentations that rank from mild to severe [17].

### Distinct mutations in the CHS domain render pyrin highly susceptible to low doses of steroid catabolites

We recently identified that the pyrin inflammasome can be activated by pregnanolone and etiocholanolone, two catabolites from the steroid hormones, progesterone and testosterone. This response is dependent on the B30.2 and on the CHS domains [12]. We thus wondered whether the identified pathogenic mutations also affected the response to steroid catabolites. U937 cell lines expressing the different *MEFV* variants were thus treated with increasing concentrations of pregnanolone and the concentration required to trigger 50% cell death (EC50) was determined. Strikingly, two mutations, p.Q426R and p.F479L, decreased pregnanolone EC50 by >50-fold (Fig. 5A). Indeed, 2 μM of pregnanolone killed 50% of p.F479L or p.Q426R-expressing cells (EC50 = 1.9 ± 2.6 μM; *n* = 5 and EC50 = 2.1 ± 3.6 μM; *n* = 6, respectively), while EC50 for WT *MEFV*-expressing cells was 110 ± 27 μM; (*n* = 7) (Fig. 5B). Similar differences were observed in response to etiocholanolone (Supplemental Fig. S5A, B) although not as drastic potentially due to the lower ability of etiocholanolone to activate the pyrin inflammasome. Cell death in all the pyrin variant-expressing cell lines was blocked by the caspase-1 inhibitor VX-765 (Fig. 5C and Supplemental Fig. S5D, E). As previously described [12], cells expressing p.M694V did not demonstrate a substantial reduction in EC50. Surprisingly, cells expressing p.H478Y variant were largely resistant to etiocholanolone (Supplemental Fig. S5A, B) and to a lower extent to pregnanolone (Fig. 5A, B). Similarly, robust secretion of cleaved caspase-1 and of cleaved GSDMD were observed in the supernatant of cell lines expressing p.Q426R and p.F479L pyrin variants treated with a low concentration of pregnanolone (6 μM, Fig. 5D). In contrast, this pregnanolone dose did not elicit detectable maturation of caspase-1 or of GSDMD in p.H478Y pyrin-expressing cells. These results suggest that mutations affecting the CHS have divergent effect on the steroid catabolites response. The same conclusions were reached when investigating the ability of pregnanolone to trigger IL-1β secretion. Indeed, IL-1β secretion rapidly decreased with decreasing doses of pregnanolone in WT or p.H478Y *MEFV*-expressing U937 cells while it was maintained until 0.2 μM in cell lines expressing p.Q426R or p.F479L *MEFV* variants (Supplemental Fig. S5F).

To get insights into the molecular mechanism, we investigated pyrin S242 phosphorylation at steady state and upon exposure to low (6 μM) or high (50 μM) doses of pregnanolone. In contrast to the high doses, low doses of pregnanolone do not promote WT pyrin dephosphorylation [12]. No major differences were seen in terms of steady state phosphorylation levels in the different cell lines (Fig. 5E). Yet, low doses of pregnanolone significantly triggered dephosphorylation of pyrin in cells expressing p.Q426R or p.F479L *MEFV* variants (Fig. 5E). In agreement with the high doses required to trigger pyrin activation in WT cells, pyrin dephosphorylation was only observed at 50 μM in the other cell lines. Similarly, UCN-01 triggered dephosphorylation in all the cell lines.

Altogether, these data indicate that distinct CHS mutations trigger distinct pyrin inflammasome deregulation. In particular, three mutations (p.Q426R, p.H478Y, p.F479L) behaved as the prototypical FMF exon 10 mutation p.M694V and rendered the pyrin inflammasome controlled only by dephosphorylation. Interestingly, only two of these (p.Q426R and p.F479L) had a further deregulation of pyrin inflammasome responses with an exquisite sensitivity to steroid catabolites.

### Primary human monocytes from patients presenting the p.F479L mutation demonstrate pyrin inflammasome response to low doses of steroid catabolites

**Fig. 6:**
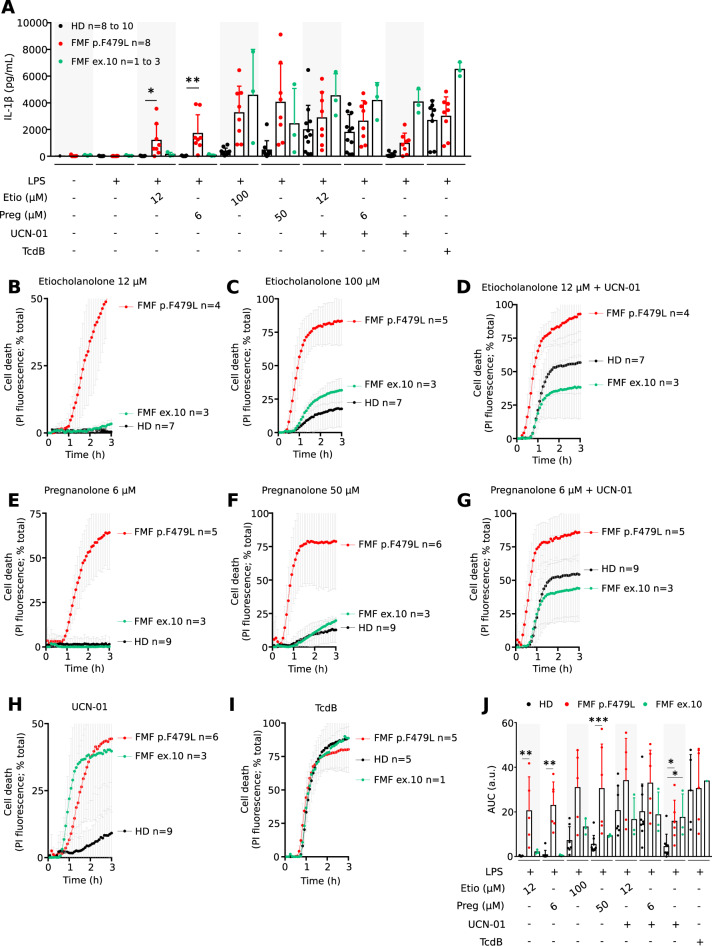
Primary human monocytes from patients presenting the p.F479L mutation demonstrated pyrin inflammasome activation in response to low doses of steroid catabolites. Monocytes from healthy donors (HD), FMF patients carrying the p.F479L mutation (see table S1 for full genotype) or FMF patients with *MEFV* exon 10 mutations were treated with indicated stimuli. **A** IL-1β concentration in the supernatant collected after 3 h of treatment was determined by ELISA. **B**–**I** PI incorporation/fluorescence was monitored every 5 min for 3 h, (**J**) corresponding AUC are shown. Data information: Mean and SD of triplicates and individual data points are shown for the indicated number of HD, FMF patients presenting p.F479L mutation and FMF patients presenting exon 10 mutation, each corresponding to the average of a biological triplicate. **J** a.u.: arbitrary units. **A** Kruskal–Wallis and Dunn’s multiple comparisons tests or (**J**) ordinary one-way ANOVA and Dunnett’s multiple comparisons tests were performed to compare the HD to the p.F479L patients. **A** *: *p* = 0.0284; **: *p* = 0.0031; (**J**) * (left to right): *p* = 0.0273, 0.0438; ** (left to right): *p* = 0.0091, 0.002; ***: *p* > 0.001.

p.Q426R and p.F479L are rare to very rare mutations (Supplemental Fig. S6A). One FMF patient with p.Q426R heterozygous mutation was identified but we could not get neither her/his clinical information nor primary cells from this patient. Eight FMF patients with the p.F479L mutation were identified in six French clinical centers (Supplemental Table S1). The p.E167D mutation co-segregates with the p.F479L mutation in a complex allele (Supplemental Fig. S6B) and was identified in at least 7 out of 8 patients. All patients were compound heterozygous patients with the p.V726 A mutation. U937 cells expressing p.E167D *MEFV* variant showed similar phenotypes as WT *MEFV*-expressing cells (Supplemental Fig. S6C–P) suggesting that this variant is likely a benign polymorphism and does not affect in a substantial way WT or p.F479L pyrin responses. p.V726A variant is usually associated with incomplete penetrance or milder forms of the disease [17]. Yet, all the patients displayed typical FMF disease (Supplemental Table S1) strongly suggesting that, in agreement with the above results and with previous studies [23, 30], the p.F479L *MEFV* variant is pathogenic. Indeed, primary monocytes from patients presenting the p.F479L mutation responded to UCN-01 by secreting IL-1β (Fig. 6A). As expected, monocytes from healthy donors did not demonstrate substantial production of IL-1β in response to UCN-01 while their pyrin inflammasome could be triggered by TcdB (Fig. 6A). We then investigated whether primary monocytes from these patients responded to low doses of the steroid catabolites pregnanolone and etiocholanolone (6 μM and 12 μM, respectively). As observed with U937 cells, primary monocytes bearing the p.F479L mutation released IL-1β in response to low doses of pregnanolone and etiocholanolone (Fig. 6A) while monocytes from HD did not. A large difference in IL-1β secretion between monocytes from HD and patient bearing the p.F479L was also seen at higher doses of steroid catabolites (50 and 100 μM) although at these doses, inflammasome activation was observed in primary monocytes from HD (Fig. 6A). Similar results were observed when investigating primary monocytes cell death in real time (Fig. 6B–J). Indeed, primary monocytes from p.F479L-expressing patients died in response to low doses of pregnanolone and etiocholanolone (Fig. 6B, E, J) in contrast to monocytes from HD. At higher doses of steroid catabolites, cell death was more extensive in primary monocytes from p.F479L-expressing patients than in monocytes from HD (Fig. 6C, F, J). As observed with IL-1β secretion (Fig. 6A), these differences were largely ablated in the presence of both steroid catabolites and UCN-01 (Fig. 6D, G, J) suggesting that the p.F479L mutation may affect a coupling mechanism between low doses of steroid catabolites and dephosphorylation. As previously described [12], FMF patients with *MEFV* mutations in exon 10 did not respond to low doses of etiocholanolone or pregnanolone but presented a moderate increase response to high doses of steroid catabolites (Fig. 6A–J).

Altogether, these results validate, in primary patients bearing the p.F479L mutation, that specific FMF-associated *MEFV* mutations render pyrin inflammasome sensitive to low doses of steroid catabolites.

## Discussion

In contrast to other inflammasome sensors such as NLRP3 [31–33], the regulation of pyrin remains unclear. Except for the phosphorylated linker domain, the roles of pyrin domains in the regulation of inflammasome activation are still largely unknown. The B30.2 domain has long been hypothesized to negatively regulate human pyrin. Yet, the proposed mechanisms are unclear and include interaction of the B30.2 with NLRP3 to mediate autophagy-mediated degradation [34], with PKN1 to modulate pyrin phosphorylation [6] or with caspase-1 [25, 26] or IL-1β [35, 36] to directly regulate these inflammasome mediators. Here, we clearly demonstrate that the B30.2 domain is dispensable for pyrin inflammasome response in response to TcdA. This result is fully in line with results obtained in mice in which pyrin is naturally devoid of the B30.2 domain [37] and with recent data obtained in U937 cells infected with *Y. pestis* [24]. Not only, the B30.2 domain is dispensable, but our data show that deletion of this domain renders the activation of the pyrin inflammasome under the sole regulation of the PKC superfamily kinases, PKN1/2. This observation thus suggests that the B30.2 domain negatively control pyrin inflammasome activation downstream of the dephosphorylation. Importantly, we observed the B30.2-mediated regulation by monitoring ASC speck formation in *CASP1*^KO^ cells. Altogether, these results demonstrate that the B30.2 regulate pyrin inflammasome activation downstream of the dephosphorylation and upstream of ASC speck formation hence regulating the step 2 of pyrin inflammasome cascade.

Surprisingly, in response to TcdA, we did not observe higher IL-1β or IL-18 responses in ΔB30.2-*MEFV*-expressing cells compared to WT *MEFV*-expressing cells while p.M694V *MEFV*-expressing cells showed higher responses. Thus, while the B30.2 deletion mimics p.M694V mutation in terms of loss of step 2 regulation, the B30.2 domain may also, at least when mutated in position p.M694V, positively regulate inflammasome activation.

The B30.2 domain sits onto a flexible coiled-coil domain that has been termed the Central Helical Scaffold (CHS). In the butyrophilin BTN31A1 protein that displays a similar domain sequence, the coiled-coil domain is implicated in the signal transduction following binding of a small ligand in the BTN3A1 B30.2 cavity. We thus made the hypothesis that the pyrin CHS domain may act together with the B30.2 domain to regulate pyrin inflammasome activation. In agreement with this hypothesis, several mutations along the CHS domains mimic p.M694V mutations and renders the pyrin inflammasome activation under the sole control of the dephosphorylation. In WT pyrin, we hypothesize that the CHS domain may transduce a negative signal initiated by the B30.2 domain. In this model, pyrin activation would be negatively regulated by these two C-terminal domains acting together to control pyrin step 2. More molecular characterization of pyrin protein and its conformational changes remains to be performed to assess the validity of this model.

While most of the research on pyrin has focused on the B30.2 domain, our results indicate that the CHS domain is a central regulator of pyrin inflammasome. Furthermore, this study and others [21, 23] demonstrate that various mutations in the CHS are pathogenic and validate that *MEFV* mutations outside exon 10 (i.e., affecting other domains than the B30.2 domain) can cause FMF. These results thus highlight the need to sequence the whole *MEFV* gene to genetically validate (or not) a clinical diagnosis of FMF.

Of particular interest, different mutations within the CHS domain have different impact on the functionality of the pyrin inflammasome in response to steroid catabolites. Particularly, two mutations (p.Q426R and p.F479L) decrease by a ≈ 50-fold the sensitivity of cells expressing pyrin to pregnanolone. Interestingly, these two mutations maps to amino-acids at the interface between two to three distinct alpha helices from two different CHS molecules within the CHS-B30.2 dimer [38]. Modifications of the CHS conformation may allow to switch the B30.2 domain from a closed to an open state [38] and the two identified mutations may thus lock or facilitate this switch.

We were not able to obtain neither primary cells nor clinical information from the single patient bearing the p.Q426R mutation described so far [28]. In contrast, we recruited 8 patients from six independent families bearing the p.F479L mutation. 7 out of 8 of these patients were (i) presenting a complex p.E167D/p.F479L allele (2) compound heterozygotes with p.V726A as the second mutation. Of note the p.V726A mutation is usually associated with mild form of FMF [17]. Except for one patient describing recurrent inflammatory flares 7 days post-menstruations, the practitioners following these patients did not report any inflammatory flares related to life events potentially associated with an increase in steroid sex hormones catabolism (puberty, pregnancy…). Yet, for 6 out of 8 patients, arthralgia/ osteoarticular manifestations were mentioned, a clinical feature previously reported in 43% of pediatric patients with two high penetrance *MEFV* mutations (p.M680I, p. M694I, p.M694V) [39]. Whether a steroid catabolite may be responsible for this clinical manifestation is unclear.

Altogether, this study demonstrates that in the C-terminus of pyrin, the B30.2 and the CHS domains regulate pyrin activation in particular by negatively regulating the step 2. *MEFV* mutations affecting the serine residues or the B30.2 domain lead to two different diseases (PAAND and FMF). Here, we identified that different mutations affecting the CHS domain differentially affect the pyrin inflammasome response and potentially the clinical presentation of the patients. Thus, our study strengthens the concept of a spectrum of *MEFV* mutations, affecting differently the regulation mechanisms of the pyrin protein and leading to Pyrin-Associated Autoinflammatory Diseases [40] with varying degrees of severity.

## Materials and methods

### Mutagenesis and cell line generation

U937 cell lines expressing the different *MEFV* variants were generated as described in Magnotti et al, 2019 [4]. Briefly, *MEFV* variants were generated by site-directed mutagenesis using the QuickChange II Site-Directed Mutagenesis Kit (Agilent) or the Q5 Site-Directed Mutagenesis Kit (NEB) with primers listed in the Supplemental Table S2. The variants were cloned into the GFP-expressing lentiviral vector pINDUCER21 [41] under a doxycycline-inducible promoter through a 3×Flag derivative of pENTR1A vector (Invitrogen). The resulting plasmids were used to produce lentiviruses in HEK293 cells. The human myeloid cell line U937 (CelluloNet, Lyon, France) was transduced with the obtained lentiviruses. Transduced cells sorted based on GFP expression on an Aria cell sorter. U937 cells were maintained in RPMI 1640 medium with glutaMAX_I supplemented with 10% FCS, 1% Hepes, 1% Sodium Pyruvate, 2mM L-glutamate, 1% PSA*. CASP1*^KO^ cell lines were generated using the CRISPR/Cas9 method as previously described [42]. HEK293T and U937 cells were tested negative for mycoplasma contamination in April 2021. The cell lines have not been authenticated.

### Cytokine detection and cell death assay

For real time cell death analysis, U937 cells were seeded at 5*10^4^ cells per well in a black 96-well plate (Costar, Corning) and treated with doxycycline (1 µg/mL) overnight. Then, the cells were stimulated with relevant compounds, propidium iodide (PI) (5 µg/mL final) was added and fluorescence measurement was immediately started and performed every 5 or 15 min for 3–16 h on a thermostatic fluorimeter (Tecan) with excitation wavelength of 535 nm (bandwidth 15 nm) and emission wavelength 635 nm (bandwidth 15 nm). The obtained results were normalized using PI incorporation values in cells treated with Triton-X100 1% v/v as 100% cell death.

To assess IL-18 secretion, U937 cells were seeded at 2*10^5^ cells per well of a 96-well plate and treated with doxycycline (1 µg/mL) overnight. The appropriate inflammasome stimuli were added and the cells were incubated at 37 °C with 5% CO_2_ for 3 h. The supernatant was collected, and IL-18 concentration was determined by ELISA with anti-human IL-18 antibody (4 µg/ml, #D044-3, MBL, Woburn, MA, USA) as capture antibody and anti-human IL-18 antibody coupled with biotin (20 ng/mL, #D045-6, MBL) as detection antibody.

To assess IL-1β secretion U937 cells were seeded at 5*10^4^ cells per well of a 96-well plate and treated with 100 ng/mL of phorbol 12-myristate 12-acetate (PMA, Invitrogen) for 3 h, then washed and incubated with 1 µg/mL doxycycline overnight. The appropriate inflammasome stimuli were added and the cells were incubated at 37 °C with 5% CO_2_ for 3 h. The supernatant was collected and the concentration in IL-1β was determined by ELISA (R&D Systems).

In each of these experiments three technical replicates were performed. The inflammasome stimuli used were: UCN-01 (12.5 µM, Sigma) without priming, TcdA at 1 µg/ml without priming and Nigericin (50 µg/mL, Invivogen) following 3 h priming with LPS (1 µg/mL, Invivogen). TcdA was purified from *Clostridioides difficile* VPI10463 strain, as previously described [43, 44].

### Immunoprecipitation and immunoblot analysis

To examine pyrin phosphorylation, U937 cells were seeded 3*10^6^ per condition in a 6-well plate and incubated with doxycycline (1 µg/mL) overnight at 37 °C with 5% CO2. The cells were then either treated or not with Carfilizomib (100 ng/mL, Selleckchem) for 30 min, then UCN-01 (12.5 µM, Sigma) for 15 min.

Once collected, the cells were washed in cold PBS and lysed in Tris HCl pH 7.0 25 mM, NaCl 150 mM, EDTA 1 mM, Igepal CA-630 (NP40) 0.1% supplemented with Sodium fluoride (phosphatase inhibitor, Sigma, S7920) at 10 μmM and, cOmplete™ EDTA-free Protease Inhibitor Cocktail (Roche 05056489001). Flag-pyrin was immunoprecipitated with Anti-FLAG® M2 Magnetic Beads (Sigma, M8823) saturated with BSA (2 mg/mL, Serva) for 2 h at 4 °C, washed in lysis buffer and denatured at 95 °C for 5 min. For Caspase-1 and GSDMD immunodetection, cells were stimulated in OptiMEM without serum for 90 min (UCN-01) to 3 h (pregnanolone). Proteins in the supernatant were precipitated by methanol-chloroform precipitation. Per lane, the supernatant from 5.10^5^ cells or 20 μg of protein lysate was loaded. Proteins were separated by SDS-PAGE on precast 4–15% acrylamide gels (Bio-Rad) and then transferred to Transblot Turbo Midi-size PVDF membranes (Bio-Rad). For immunoblotting the following antibodies were used: mouse anti-Flag (Sigma Aldrich, clone M2, 1:1000), rabbit anti-Pyrin (Adipogen, AL196, 1:1,000), rabbit anti-phosphoS241 Pyrin (Abcam, ab200420, 1:1,000), mouse anti-V5 tag (Invitrogen, 37–7500, 1:1,000), rabbit anti-Caspase-1 (#3866 S, Cell signaling, 1:1,000), rabbit anti-GSDMD (#HPA044487, Sigma, 1:1,000), mouse anti-βActin (Sigma Aldrich, clone C4, MAB1501, 1:5,000) and anti-mouse IgG (Promega, W4021, 1:3,000) and anti-rabbit IgG (Sigma, A0545, 1:3,000).

Figure panel 2D was performed using an automated capillary-based Western blot system (Jess, Biotechne) using a 12–230 KDa separation module (SM-W004, Biotechne). 3 μL of cell lysate at 2 mg/mL was loaded in each well. For cell supernatant samples, the medium was changed at 3 h post-doxycycline addition to optiMEM. 6 h later (9 h post-doxycycline addition), supernatant was collected and precipitated by chloroform-methanol. 3 μL of cell supernatant -corresponding to 3.5 10^4^ cells- was loaded in each well. GSDMD antibody (#HPA044487, Sigma) was multiplexed with actin antibody (#MAB1501, Sigma), both used at 1:50 dilution and detected respectively in chemiluminescence and fluorescence using anti-rabbit HRP antibody (#DM-001, Biotechne) and anti-Mouse NIR antibody (#DM-009, Biotechne).

Uncropped Western blot images are available in the Supplemental material.

### Immunofluorescence

To quantify ASC specks, U937 cells were seeded 2*10^5^ per condition in a 96-well V-bottom plate and incubated with or without doxycycline (1 µg/mL) overnight. Then the cells were additionally treated or not with UCN-01 12.5 µM for 25 min, washed with PBS, and fixed with 2% paraformaldehyde (15 min at 37 °C). The cells were washed once in PBS and transferred onto glass slides with cytospin, then permeabilized with Triton-X100 0.1%v/v in PBS. The samples were blocked in PBS, BSA (3%, Serva), Triton X-100 (1%, Sigma), stained with anti-ASC (Santa Cruz, sc22514R, 4 µg/mL in blocking buffer) for 1 h at room temperature, then with donkey anti-rabbit Alexa Fluor 594 (Life Technologies, A21207, 10 µg/mL), followed by DAPI (100 ng/ml). Coverslips were mounted using mowiol.

### Quantification and statistical analysis

Normality was verified using D’Agostino & Person omnibus normality test, Shapiro–Wilk normality test or Kolmogorov–Smirnov test with Dallal-Wilkinson-Lille for *P* value if the number of values was too small for the former test. Gaussian distribution was assumed for technical triplicates. Normal unmatched values were analyzed with one-way ANOVA, with Dunnett’s correction for multiple comparisons. Unmatched values, for which normality could not be verified, were analyzed using Kruskal–Wallis analysis with Dunn’s correction. Equal variance was not assumed. Prism 7 (GraphPad) was used for statistical analyses. The statistical analyses and parameters for each experiment are listed in the corresponding figure legends.

## Supplementary information

Supplemental Table S1

Supplemental Table S2

Supplemental figures legend

Supplementary Figures S1-S6

Uncropped Western Blots

Manuscript

Cdd author contribution form

## Data Availability

Further information and requests for resources and reagents should be directed to the lead contact, TH (thomas.henry@inserm.fr). All unique/sable reagents generated in this study are available from the lead contact without restriction. Raw data (full Western blot images and Raw data) have been deposited at Mendeley (doi: 10.17632/22m9h3243f.1). This paper does not report original code. Any additional information required to reanalyze the data reported in this paper is available from the lead contact upon request.
