# Supplementary material for: Mutations in the B30.2 and the central helical scaffold domains of pyrin differentially affect inflammasome activation
Source: Cell Death Dis. 2023 Mar 25;14(3):213. doi: 10.1038/s41419-023-05745-9 (PMC10039897; doi:10.1038/s41419-023-05745-9)
Supplement: Supplementary file 1 — Supplemental Table S1 [file 41419_2023_5745_MOESM1_ESM.docx]

**Supplemental material:**

| # | Sex | Mutation^1^ | Age | Age onset | Colchicine treatment | Symptoms |
| --- | --- | --- | --- | --- | --- | --- |
| P1 | F | p.E167D, p.F479L, p.V726A | 35 | 13 | 2 mg/ day | Mild hidradenitis suppurativa disease.  3-day episodes of abdominal pain with arthralgia |
| P2 | F | p.E167D, p.F479L, p.V726A | 41 | 10 | 1 mg / day | 3-day episodes of abdominal pain with arthralgia |
| P3 | M | p.E167D, p.F479L, p.V726A | 67 | 24 | 1 mg / day | Skin rash, abdominal pain, joint pain, total hip replacement, occasional fatigue, or physical weakness |
| P4 | F | p.E167D, p.F479L, p.V726A | 11 | 2.5 | 0.5 mg /day | Variable proteinuria and some leucorrhea, aseptic long-lasting osteoarticular manifestations, regress with colchicine, no biological inflammatory syndrome |
| P5 | M | p.E167D, p.F479L, p.V726A | 10 | 2 | 0.5 mg /day | Occasional aseptic osteoarticular manifestations, regress spontaneously or with colchicine, no biological inflammatory syndrome |
| P6 | M | p.E167D, p.F479L, p.V726A | 7 | 2.5 | 0.5 mg /day | Occasional aseptic osteoarticular manifestations, regress spontaneously or with colchicine, persistent cervical adenopathy, biological inflammatory syndrome |
| P7 | F | p.F479L, p.V726A^2^ | 32 | 8-10 | 1 mg / day | 3 day episodes of abdominal pain with arthralgia; frequently 7 days post-menstruation |
| P8 | M | p.E167D, p.F479L, p.V726A | 11 | 4 | 1 mg / day | Typical FMF phenotype |

**Table S1. Clinical profiles of FMF patients with the p.F479L *MEFV* mutation (related to figure 6).**

^1^ p.E167D and p.F479L are likely on the same complex allele although this was not specifically tested in these patients.

^2^ Patient P7 had been genotyped out of the framework of this study. It is unclear if she may also present the p.E167D mutation
