# Supplementary material for: Mutations in the B30.2 and the central helical scaffold domains of pyrin differentially affect inflammasome activation
Source: Cell Death Dis. 2023 Mar 25;14(3):213. doi: 10.1038/s41419-023-05745-9 (PMC10039897; doi:10.1038/s41419-023-05745-9)
Supplement: Supplementary file 2 — Supplemental Table S2 [file 41419_2023_5745_MOESM2_ESM.docx]

| **Table S2.** Site-directed mutagenesis primers | |
| --- | --- |
| M694V - WT | 5'-GGTACTCATTTTCCTTCATCATTATCACCACCCAGTAG-3' |
|  | 5'-CTACTGGGTGGTGATAATGATGAAGGAAAATGAGTACC-3' |
| WT – Δ1-92 | 5’ – GAATATTCCACACAAGAAAACGGCACAGATG – 3’ |
|  | 5’ – CGCGGCCGCAAGCTTGTC – 3’ |
| WT – Δ88-310 | 5’ – GAAGGCCACCAGACACGG – 3’ |
|  | 5’ – CCTGAATGGCTGCCCTGT – 3’ |
| WT – Δ371-412 | 5’ – GGGGCTTAGGCTTCCCGGGCGAGGAGGTCGCCCTGGAACA – 3’ |
|  | 5’ – GCCCGGGAAGCCTAAGCCCCTGTTCCAGGGCGACCTCCTC – 3’ |
| WT – Δ421-440 | 5’ – CGATCCTATGGGGAGGAG – 3’ |
|  | 5’ – CTTGTGTTCCAGGGCGAC – 3’ |
| WT – S580X | 5’ – GAACATTTCCATTTCTTAACGCAGGGTTTCTGAGAAGTAC – 3’ |
|  | 5’ – GTACTTCTCAGAAACCCTGCGTTAAGAAATGGAAATGTTCAA – 3’ |
| WT - S242R | 5'- GAAATGGTGACCTCAAGTCTTCTAGGTCGCATCTT-3' |
|  | 5'- AAGATGCGACCTAGAAGACTTGAGGTCACCATTTC-3' |
| WT - S208C | 5'- CCCCGCGGAGCAGGCGTTTCTGC-3' |
|  | 5'- GCAGAAACGCCTGCTCCGCGGGG-3' |
| WT – Q426R | 5’ – AAGAAGAAAATTCAGAAGCGGCTGGAGCATCTGAAGAAG - 3’ |
|  | 5’ - CTTCTTCAGATGCTCCAGCCGCTTCTGAATTTTCTTCTT - 3’ |
| WT – H478Y | 5’ - CCTGGAGCAGCAAGAGTATTTCTTTGTGGCCTC - 3’ |
|  | 5’ - GAGGCCACAAAGAAATACTCTTGCTGCTCCAGG - 3’ |
| WT – F479L | 5’ - GAGCAGCAAGAGCATTTGTTTGTGGCCTCACTGG - 3’ |
|  | 5’ - CCAGTGAGGCCACAAACAAATGCTCTTGCTGCTC - 3’ |
| WT – E552D | 5’ - GTGGACCACTCCTCAAGACATAAAACAAAAGATCCAAC - 3’ |
|  | 5’ - GTTGGATCTTTTGTTTTATGTCTTGAGGAGTGGTCCAC - 3’ |
| WT – L559F | 5’ - CCTCAAGAGATAAAACAAAAGATCCAATTCCTCCACCAGAAG - 3’ |
|  | 5’ - CTTCTGGTGGAGGAATTGGATCTTTTGTTTTATCTCTTGAGG - 3’ |
| WT – E167D | 5’ - GTCCAGGCCGTCCGAGGCCTTCTCTCT - 3’ |
|  | 5’ - AGAGAGAAGGCCTCGGACGGCCTGGAC - 3’ |
