# Supplementary material for: Mutations in the B30.2 and the central helical scaffold domains of pyrin differentially affect inflammasome activation
Source: Cell Death Dis. 2023 Mar 25;14(3):213. doi: 10.1038/s41419-023-05745-9 (PMC10039897; doi:10.1038/s41419-023-05745-9)
Supplement: Supplementary file 3 — Supplemental figures legend [file 41419_2023_5745_MOESM3_ESM.pdf]

Supplemental figure legends

Figure S1. TcdA-mediated inflammasome activation is dependent on doxycycline-mediated induction of pyrin variants (related to Fig. 1). U937 monocytes (B) or macrophages incubated in the absence of doxycycline, were treated with TcdA for 6 h or LPS for 3 h followed by Nigericin for 3 hours. The concentration of IL-1β (A) and IL-18 (B) in the supernatant was determined by ELISA. Results in the presence of doxycycline are presented in Fig. 1.

Figure S2. UCN-01-mediated cell death is dependent on doxycycline-mediated induction of pyrin variants and blocked by a caspase-1 inhibitor (related to Fig. 2). U937 monocytes (B) or macrophages incubated in the absence of doxycycline, were treated with UCN-01 for 2 h or LPS for 3 h followed by Nigericin for 3 hours. The concentration of IL-1β (A) and IL-18 (B) in the supernatant was determined by ELISA. Results in the presence of doxycycline are presented in Fig. 2. (C) U937 monocytes expressing the indicated variants under the control of a doxycycline inducible were treated with doxycycline in the presence or not of the caspase-1 inhibitor, VX-765. Propidium iodide incorporation was monitored every 15 min for 16 h. Cell death was calculated following normalization with TX-100-treated cells (100%) and cells cultivated in the absence of doxycycline (0%). (D) The corresponding area under the curve (AUC) expressed in arbitrary units (a.u.) are shown. One-way ANOVA with Sidak's correction for multiple comparisons test was performed. ***: $p<0.001$.

Figure S3. Expression of pyrin in *Caspase-1*^{KO} U937 monocytes and IL-18 secretion in response to UCN-01 and nigericin. U937 monocytes knocked-out or not for *CASP1* expressing the indicated pyrin variants under the control of a doxycycline-inducible promoter were generated. (A) Pyrin expression was verified by Western blot. The monocytes were treated with (B) UCN-01 and (C) Nigericin, IL-18 concentration was measured in the supernatant with ELISA.

Figure S4. Expression of pyrin variants mutated in the CHS domain and impact of the CHS mutations on NLRP3 activation. U937 monocytes expressing the indicated pyrin variants under the control of a doxycycline-inducible promoter were generated. (A) Pyrin expression was verified by Western blot. (B-D) The indicated cell lines were treated with LPS for 3 h, then with Nigericin. (B) Cell death kinetics were monitored through PI/fluorescence every 5 minutes over the indicated time, (C) corresponding AUC are shown. (D) IL-1β concentration in the supernatant was measured by ELISA.

Data information: Data from one experiment representative of three independent experiments. Mean and SD of triplicates and individual data points are shown. a.u.: arbitrary units. (C) Kruskal-Wallis and Dunn's multiple comparisons tests or (D) ordinary one-way ANOVA and Dunnett's multiple comparisons tests were performed to compare U937 cells expressing WT pyrin to those expressing other variants. (D) ***: $p<0.001$

Figure S5. Mutations in the CHS domain of pyrin affect caspase-1 mediated cell death and lead to differential IL-1β secretion in response to the steroid catabolites, etiocholanolone and pregnanolone.

U937 monocytes incubated with doxycycline (unless otherwise indicated (C)) to induce expression of the indicated pyrin variants, were treated (A-B) with decreasing doses of etiocholanolone or (C-E) the indicated stimuli. (A-E) Cell death levels were measured at 3 h post-treatment. (B) EC50 was determined at 3 h post-treatment. (C) Cell death levels were measured at 3 h post-treatment in the absence of doxycycline or in the presence of VX-765. Results were normalized to the cell death value of cells treated with doxycycline and etiocholanolone at 100 μM. (D-E) Real time cell death curves corresponding to Fig. 5C and Fig. S5C. (F) U937 monocytes expressing the indicated pyrin variants were treated with decreasing doses (5-fold dilution) of pregnanolone ranging from 500 μM to 0.16 μM. IL-1β concentration in the supernatant was measured by ELISA.

Data information: (A) Data from one experiment representative of three independent experiments. The curve was obtained by an ordinary (Least squares) fit using the log (agonist) vs. normalized response-variable slope model. (B) EC values were obtained from the above analysis using best-fit values. Each point represents the mean EC50 calculated from one biological triplicate. The bars represent the mean and SD of 3 to 7 independent experiments. Ordinary one-way ANOVA and Dunnett's multiple comparisons tests were performed. (C) Data from one experiment. Each point represents the value of one well, mean and SEM are shown. (D-E) Data from one experiment. (B) Ordinary one-way ANOVA and Dunnett's multiple comparisons tests were performed. **: p=0.0022; *: p=0.0405. (C) One way Anova with Sidak's correction for multiple test was applied. Two-tailed p-values are shown. ***: p<0.001. (F) Data from one experiment representative of two independent experiments. Mean and SD of triplicates and individual data points are shown. Kruskal-Wallis and Dunn's multiple comparisons tests or ordinary one-way ANOVA and Dunnett's multiple comparisons tests for U937 monocytes expressing the p.L559F variant of pyrin were performed to compare IL-1β secretion induced by 500 µM of pregnanolone to that induced by each of the smaller doses. * (left to right): p=0.0329, 0.0187; ** (left to right): p=0.0016, 0.0028, 0.0041; ***: p>0.001.

Figure S6. p.F479L variant drives the pyrin hyper-responsiveness in cells expressing pyrin complex allele with the two mutations [p.E167D; F479L]. (A) Frequency of p.Q426R and p.F479L variants as compared to the PAAND and the most frequent FMF-associated pyrin mutations according to the Genome Aggregation Database (gnomAD) (B) Frequency of co-occurrence of p.E167D and p.F479L pyrin variants according to gnomAD. (C) U937 monocytes expressing the indicated pyrin variants under the control of a doxycycline-inducible promoter were generated. These cell lines were treated as indicated. (D-G) IL-1β concentration in the supernatant was determined by ELISA. (H, J, L-M, O) Cell death kinetics were monitored through PI incorporation/fluorescence every 5 minutes over the indicated time. (I, K, N, P) Corresponding AUC are shown.

Data information: (D-P) Data from one experiment representative of three independent experiments. Mean and SD of triplicates and individual data points are shown. (I, K, N, P) a.u.: arbitrary units. (D-F, I, K, N, P) Ordinary one-way ANOVA and Dunnett's multiple comparisons tests or (G) Kruskal-Wallis and Dunn's multiple comparisons tests were performed to compare U937 cells expressing WT pyrin to those expressing other variants.

(D) ***: $p<0.001$; (E) **: $p=0.0018$; ***: $p<0.001$; (F) ***: $p<0.001$; (I) **: $p=0.0033$; ***:$p<0.001$; (K) **: $p=0.0011$; ***: $p<0.001$; (N) ** (left to right): $p=0.0064$, 0.0038; ***: $p<0.001$
