# Supplementary figures and images for: Mutations in the B30.2 and the central helical scaffold domains of pyrin differentially affect inflammasome activation
Source: Cell Death Dis. 2023 Mar 25;14(3):213. doi: 10.1038/s41419-023-05745-9 (PMC10039897; doi:10.1038/s41419-023-05745-9)

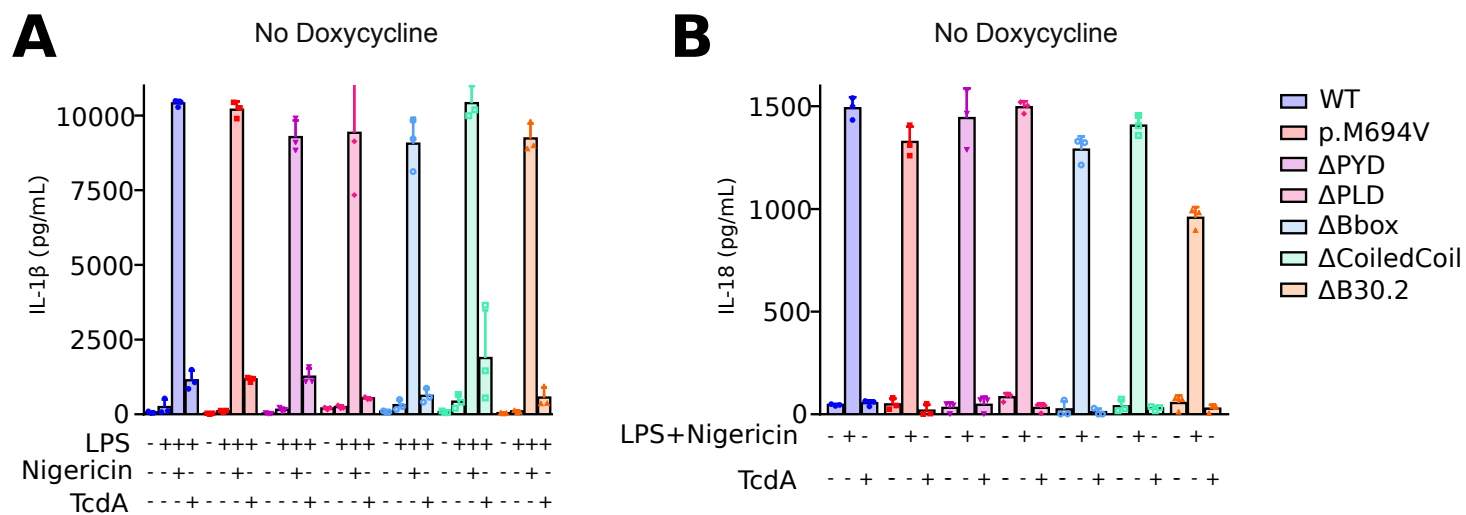

Figure S1



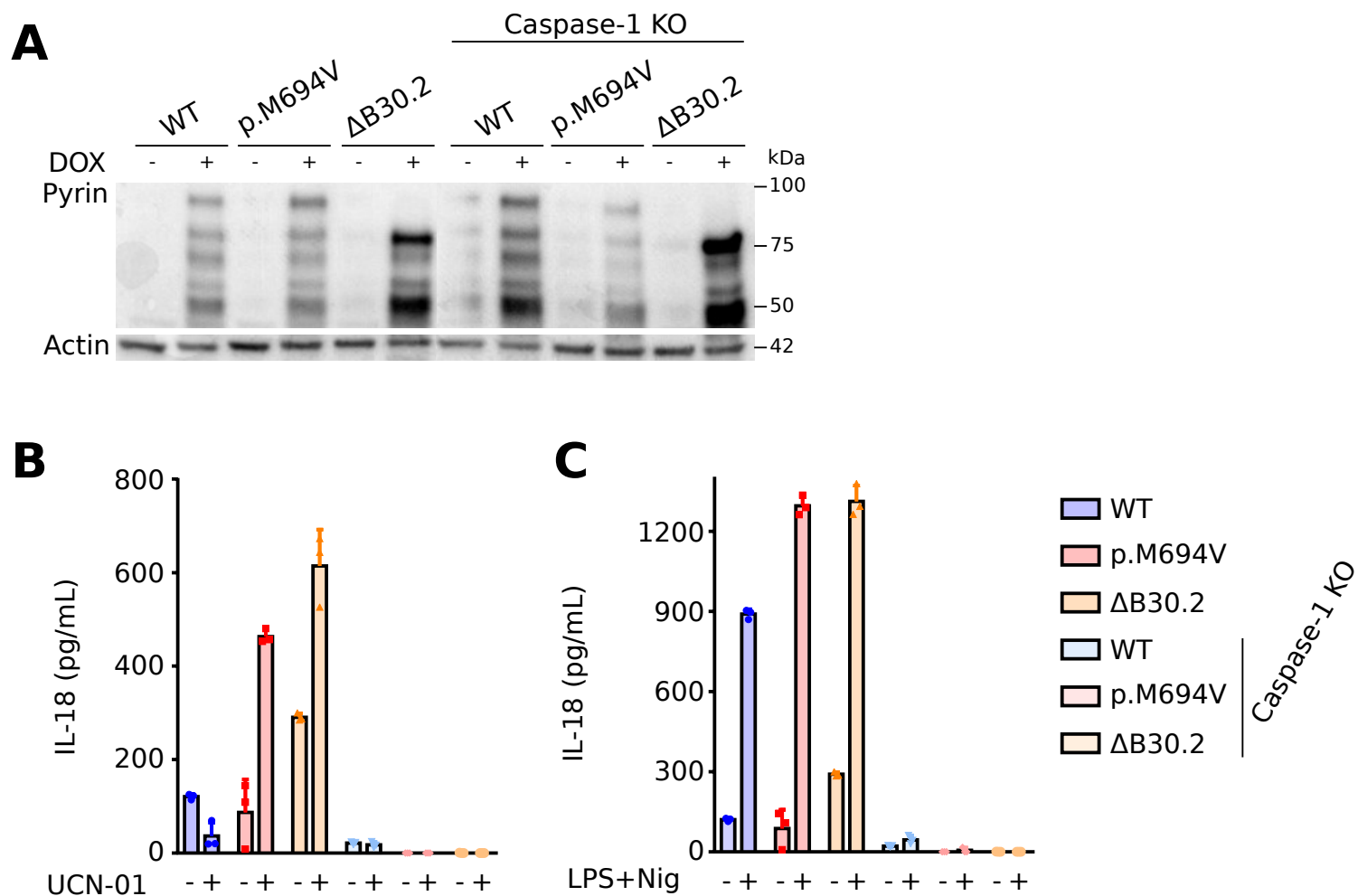

Figure S3

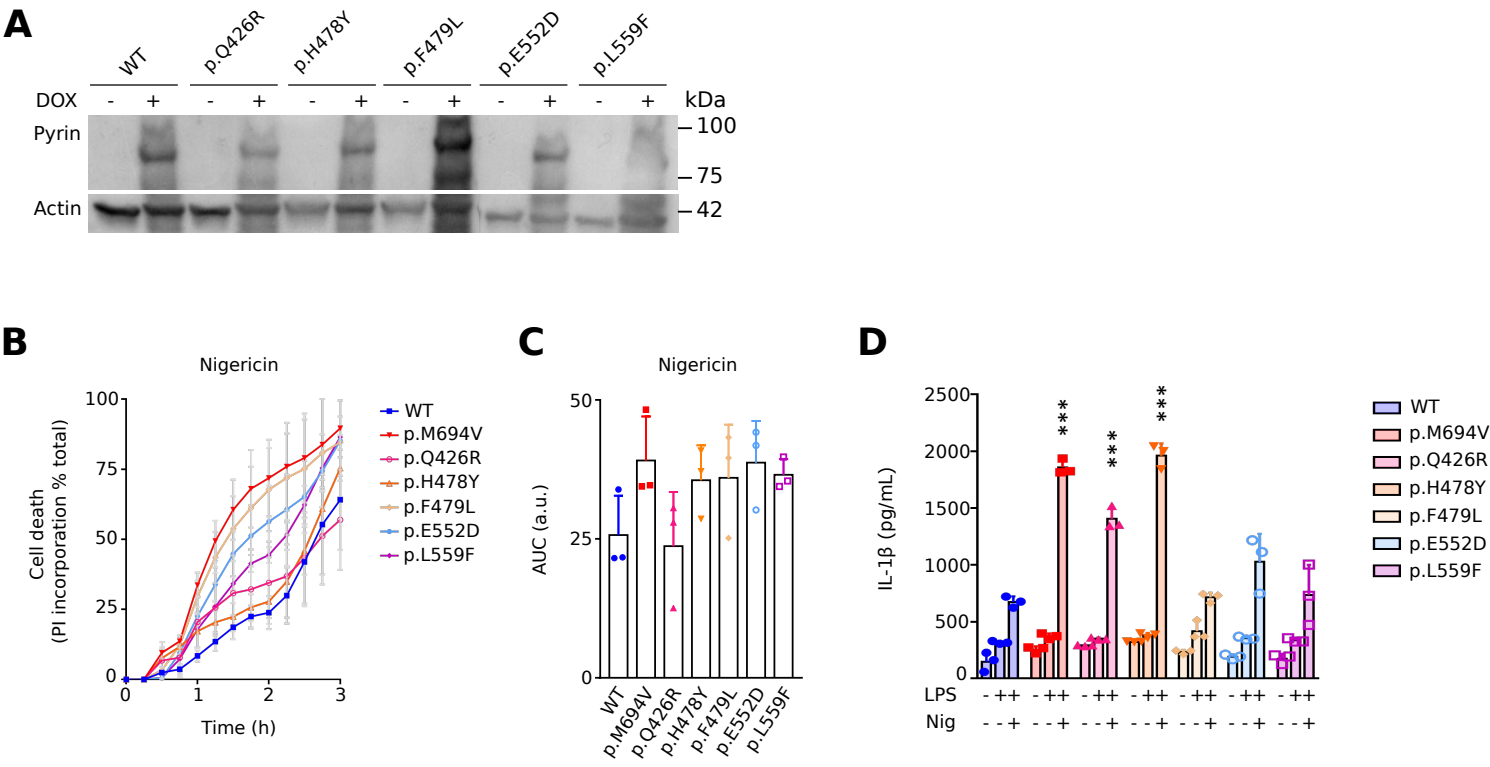

Figure S4

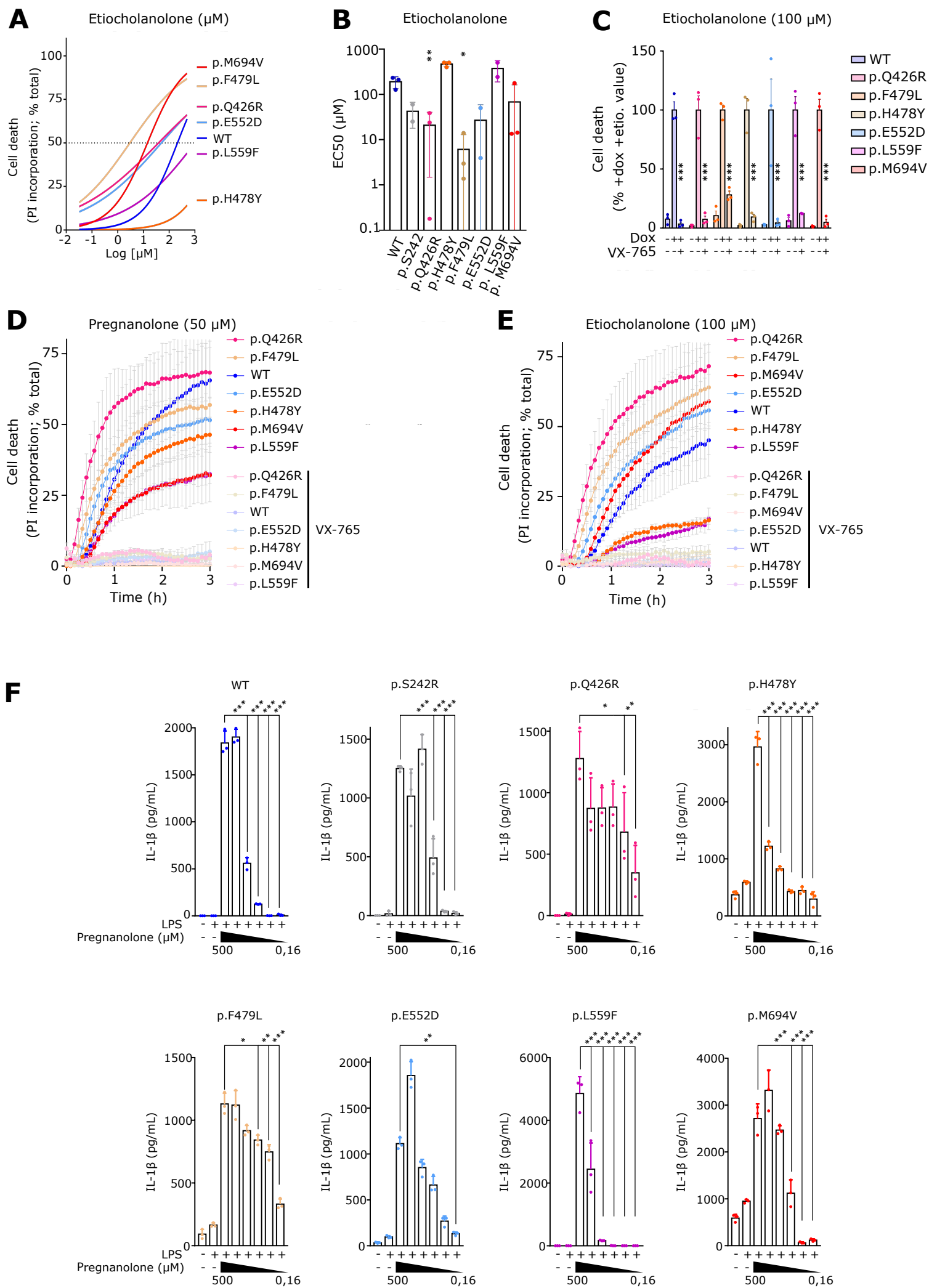

Figure S5

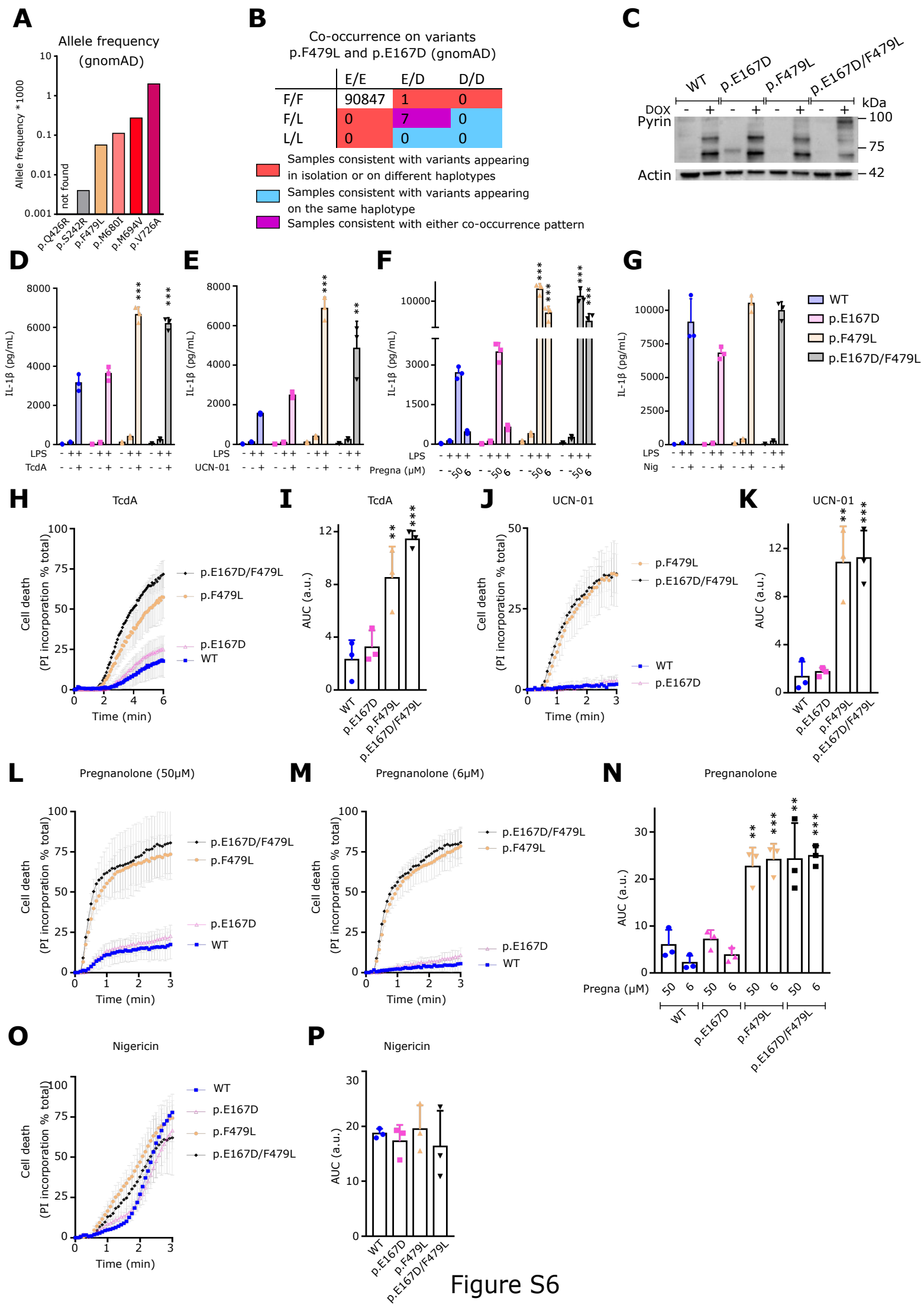

Figure S6

Supplement: Supplementary file 4 — Supplementary Figures S1-S6 [file 41419_2023_5745_MOESM4_ESM.pdf]
