# Supplementary material for: Mutations in the B30.2 and the central helical scaffold domains of pyrin differentially affect inflammasome activation
Source: Cell Death Dis. 2023 Mar 25;14(3):213. doi: 10.1038/s41419-023-05745-9 (PMC10039897; doi:10.1038/s41419-023-05745-9)
Supplement: Supplementary file 5 — Uncropped Western Blots [file 41419_2023_5745_MOESM5_ESM.pdf]

Lysate

Supernatant

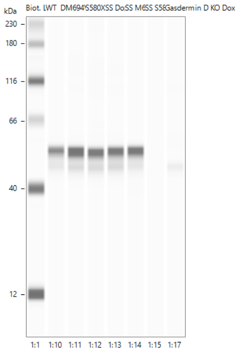


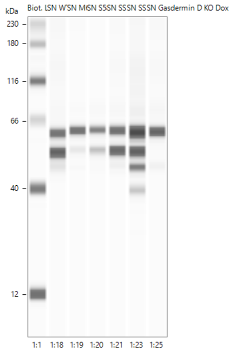


GSDMD

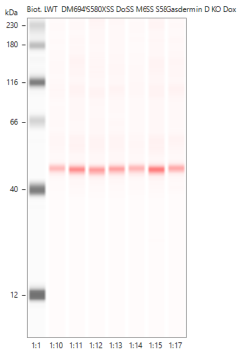


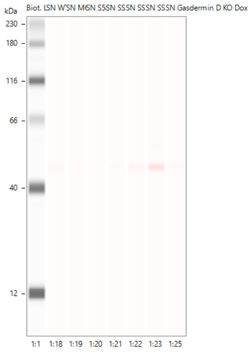


Actin

Supplementary file 1 (related to Figure 2D)

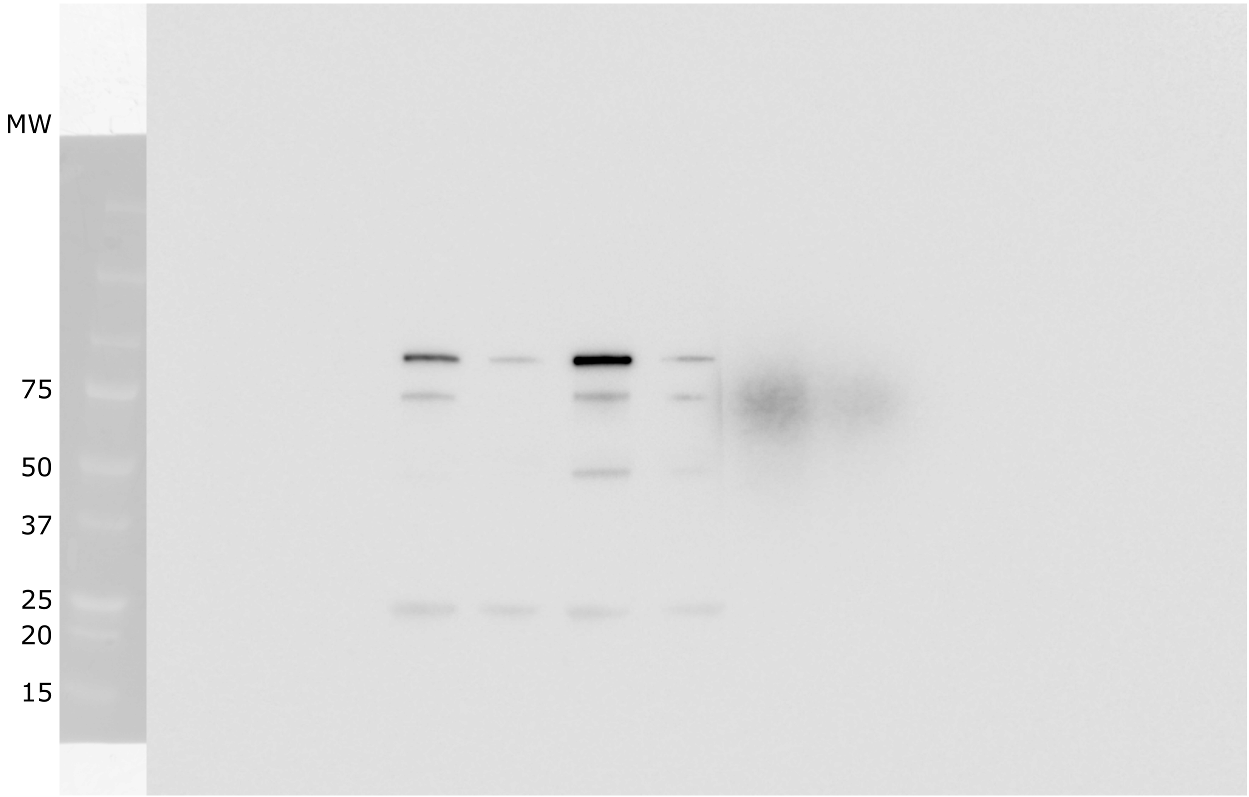


IP Flag, IB pyrin p-S242 ~ 80 kDa. Samples (left to right): pyrin WT untreated, pyrin WT + UCN-01, pyrin p.M694V untreated, pyrin p.M694V + UCN-01. Exposure 60 seconds.

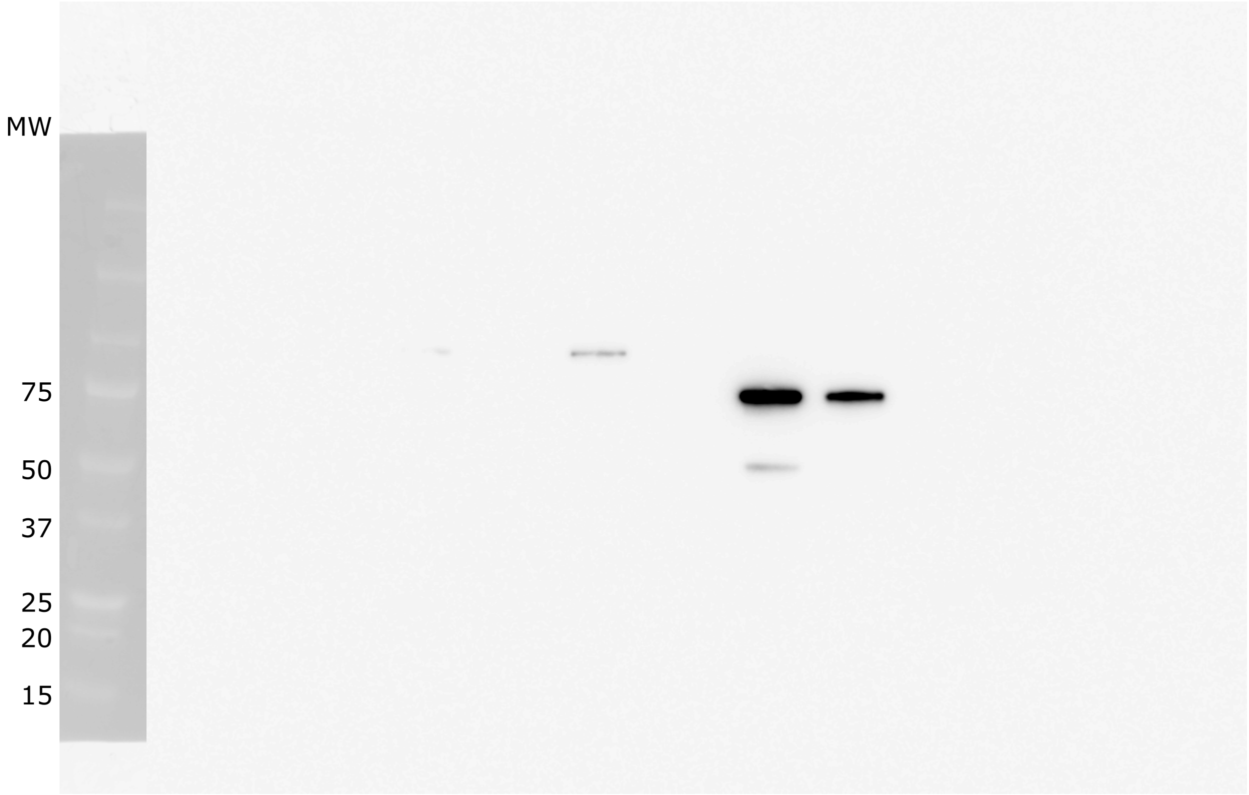


IP Flag, IB pyrin p-S242 ~ 80 kDa. Samples (left to right): pyrin ΔB30.2 untreated, pyrin ΔB30.2 + UCN-01. Exposure 20 seconds.

Supplementary file 2 (related to Figure 3A)

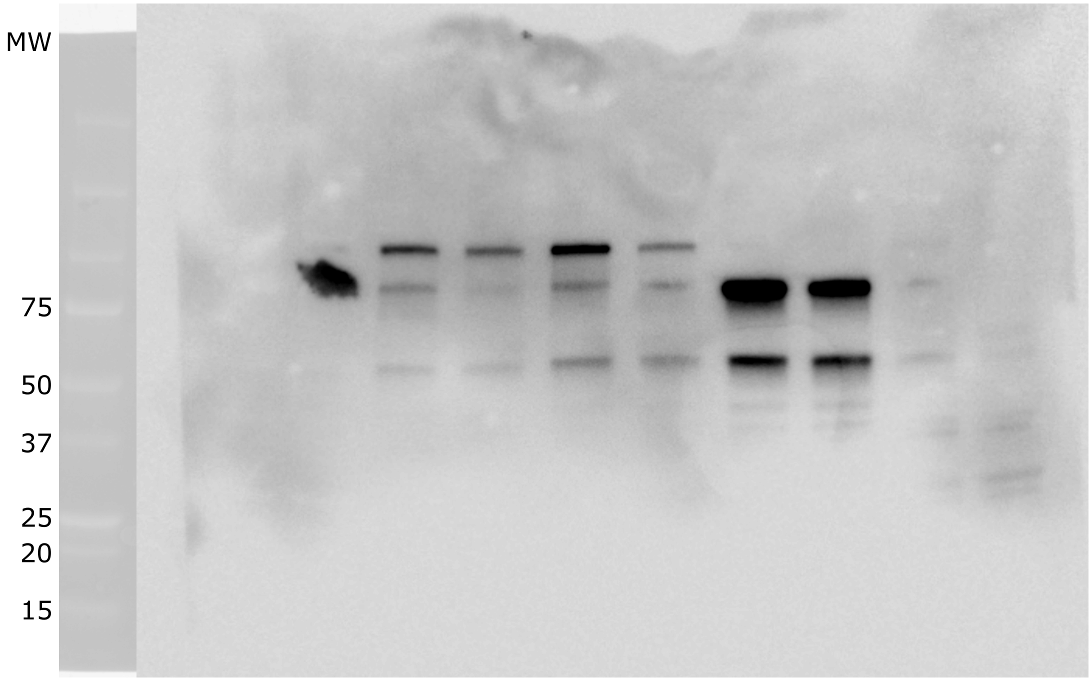


IP Flag, IB pyrin ~ 80 kDa. Samples (left to right): pyrin WT untreated, pyrin WT + UCN-01, pyrin p.M694V untreated, pyrin p.M694V + UCN-01, pyrin ΔB30.2 untreated, pyrin ΔB30.2 + UCN-01. Exposure 60 seconds.

Supplementary file 3 (related to Figure 3A)

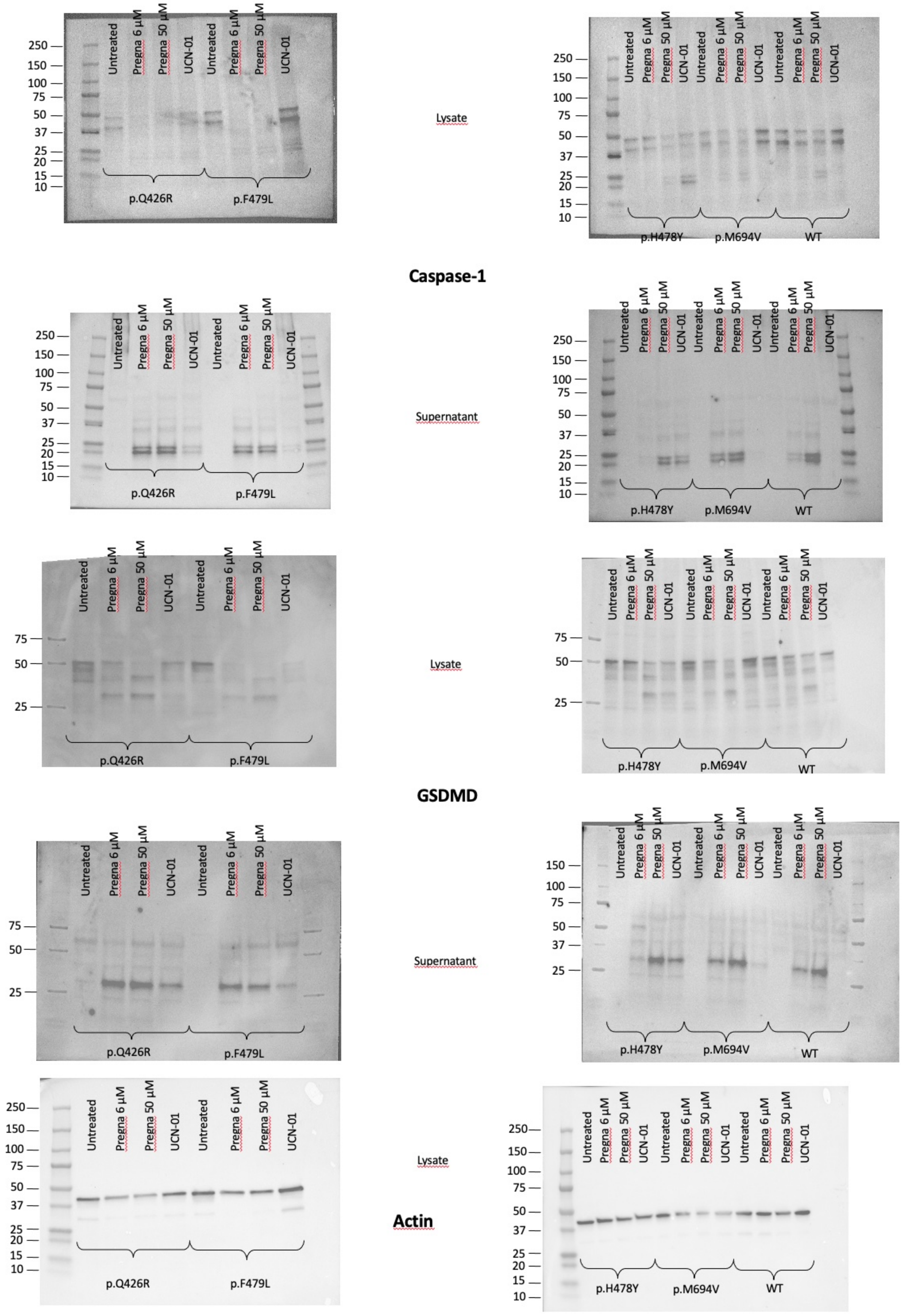


Supplementaty file 4 (related to Fig. 5D)

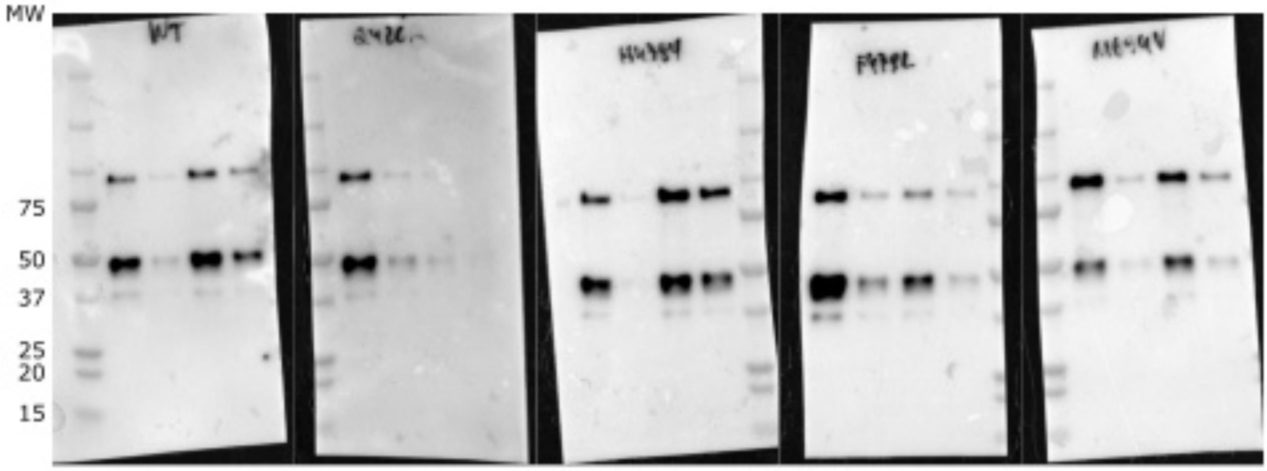


IP Flag, IB pyrin p-S242 ~ 80 kDa. Samples (left to right): pyrin WT: untreated, + UCN-01, + Pregnanolone 6 μM, + Pregnanolone 50 μM; pyrin p.Q426R: untreated, + UCN-01, + Pregnanolone 6 μM, + Pregnanolone 50 μM; pyrin p.H478Y: untreated, + UCN-01, + Pregnanolone 6 μM, + Pregnanolone 50 μM; pyrin p.F479L: untreated, + UCN-01, + Pregnanolone 6 μM, + Pregnanolone 50 μM; pyrin p.M694V: untreated, + UCN-01, + Pregnanolone 6 μM, + Pregnanolone 50 μM.

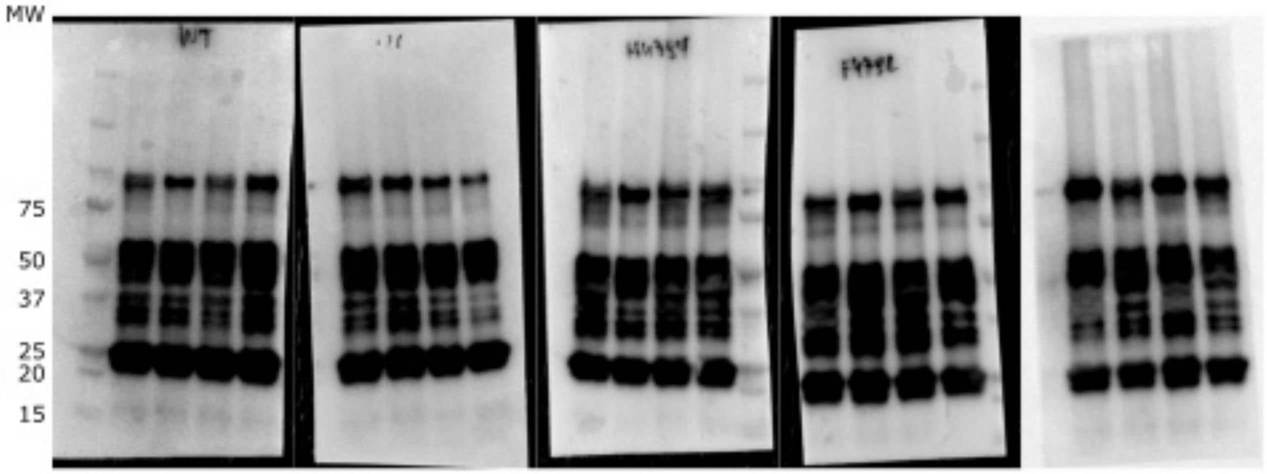


IP Flag, IB Flag ~ 80 kDa. Samples (left to right): pyrin WT: untreated, + UCN-01, + Pregnanolone 6 μM, + Pregnanolone 50 μM; pyrin p.Q426R: untreated, + UCN-01, + Pregnanolone 6 μM, + Pregnanolone 50 μM; pyrin p.H478Y: untreated, + UCN-01, + Pregnanolone 6 μM, + Pregnanolone 50 μM; pyrin p.F479L: untreated, + UCN-01, + Pregnanolone 6 μM, + Pregnanolone 50 μM; pyrin p.M694V: untreated, + UCN-01, + Pregnanolone 6 μM, + Pregnanolone 50 μM.

Supplementary file 5 (related to Figure 5E)

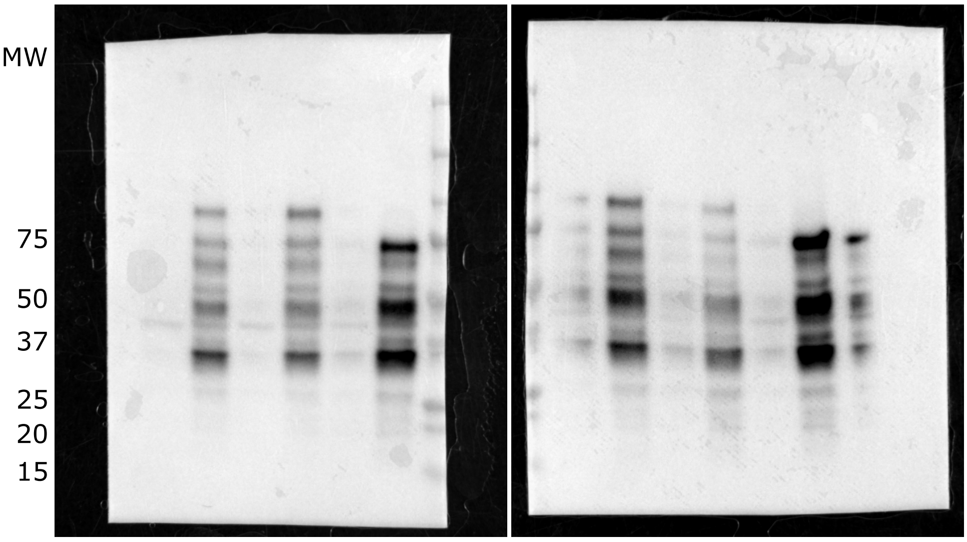


IB Flag ~ 80 kDa. Samples (left to right): pyrin WT: untreated, + dox; pyrin p.M694V: untreated, + dox; pyrin ΔB30.2: untreated, + dox; Caspase-1 KO pyrin WT: untreated, + dox; Caspase-1 KO pyrin p.M694V: untreated, + dox; Caspase-1 KO pyrin ΔB30.2: untreated, + dox.

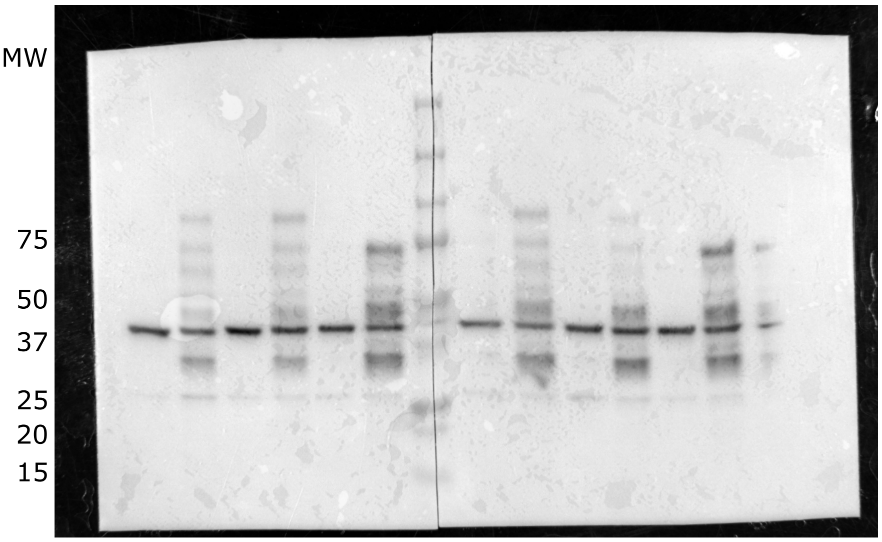


IB Actin ~ 42 kDa. Samples (left to right): pyrin WT: untreated, + dox; pyrin p.M694V: untreated, + dox; pyrin ΔB30.2: untreated, + dox; Caspase-1 KO pyrin WT: untreated, + dox; Caspase-1 KO pyrin p.M694V: untreated, + dox; Caspase-1 KO pyrin ΔB30.2: untreated, + dox.

Supplementary file 6 (related to Figure S3A)

MW

75

50

37

25

20

15

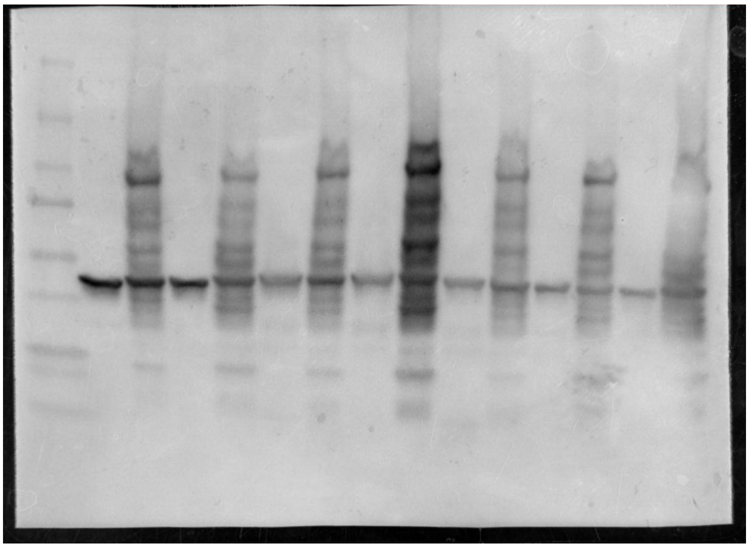

IB Flag ~ 80 kDa and Actin ~ 42 kDa. Samples (left to right): pyrin WT: untreated, + dox; pyrin p.Q426R: untreated, + dox; pyrin p.H478Y: untreated, + dox; pyrin p.F479L: untreated, + dox; pyrin p.E552D: untreated, + dox; pyrin p.L559F: untreated, + dox.

Supplementary file 7 (related to Figure S4A)

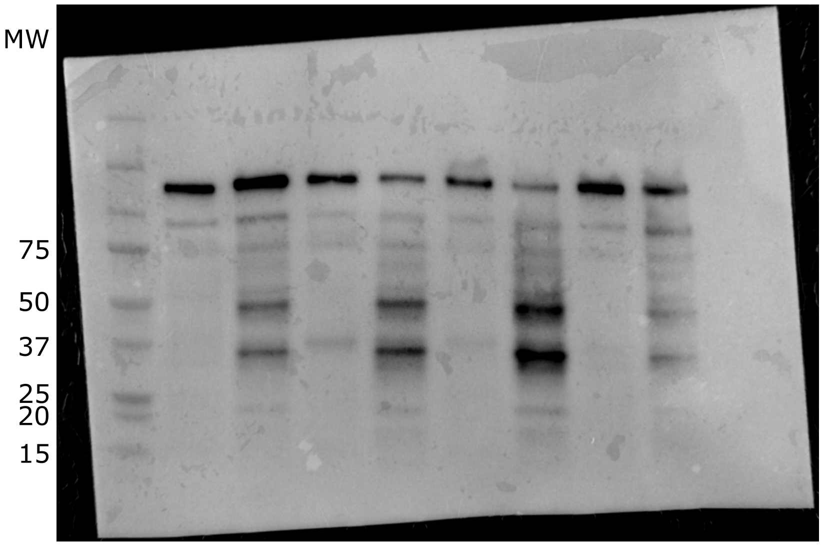


IB Flag ~ 80 kDa. Samples (left to right): pyrin WT: untreated, + dox; pyrin p.E167D: untreated, + dox; pyrin p.F479L: untreated, + dox; pyrin p.E167D/F479L: untreated, + dox.

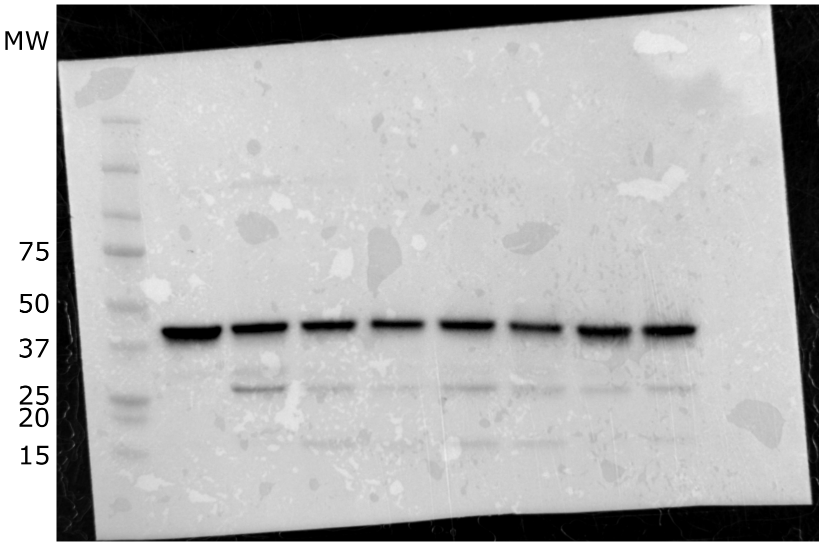


IB Actin ~ 42 kDa. Samples (left to right): pyrin WT: untreated, + dox; pyrin p.E167D: untreated, + dox; pyrin p.F479L: untreated, + dox; pyrin p.E167D/F479L: untreated, + dox.

Supplementary file 8 (related to Figure S6C)
