# Supplementary material for: Mutations in the B30.2 and the central helical scaffold domains of pyrin differentially affect inflammasome activation
Source: Cell Death Dis. 2023 Mar 25;14(3):213. doi: 10.1038/s41419-023-05745-9 (PMC10039897; doi:10.1038/s41419-023-05745-9)
Supplement: Supplementary file 7 — Cdd author contribution form [file 41419_2023_5745_MOESM7_ESM.pdf]

DECLARATION OF CONTRIBUTIONS TO ARTICLE

ADMC

Manuscript Number: CDD-22-2343

Journal Name: *Cell Death & Differentiation* (the 'Journal')

Proposed Title of the Contribution:

Mutations in the B30.2 and the Central Helical scaffold domains of pyrin differentially affect inflammasome activation (the 'Contribution)

Author(s):

Daria Chirita1, Flora Magnotti1, Pauline Bronnec1, Sarah Dalmon1, Amandine Martin1, Michel Popoff2, Mathieu Gerfaud-Valentin3, Pascal Sève3, Alexandre Belot1,4,5, Anne Contis6, Agnes Duquesne5, Gaetane Nocturne7, Irene Lemelle8, Sophie Georgin-Lavialle9, Guilaine Boursier10, Isabelle Touitou10, Yvan Jamilloux1,3,4, Thomas Henry (the 'Authors')

For all *CDD* articles, each person named as an author in the published version must be able to show he or she has contributed substantially to the article.

Authorship credit should be based on 1) substantial contributions to conception and design, acquisition of data, or analysis and interpretation of data; 2) drafting the article or revising it critically for important intellectual content; and 3) final approval of the version to be published. Authors should meet conditions 1, 2 and 3.

Any person who cannot be shown to have made a substantial contribution to the article cannot be listed as an author in the final version. The name of any person who is deemed to have made a minor contribution can, however, appear in the Acknowledgments section of the article.

Please complete the table below to indicate the contributions of all named authors to the manuscript.

Author Full Name:	Specification of Contribution to the Manuscript:
Daria Chirita	Acquisition, analysis of data + draft + final Approval
Flora Magnotti	Acquisition, analysis of data + final Approval
Pauline Bronnec	Acquisition, analysis of data + final Approval
Sarah Dalmon	Acquisition, analysis of data + final Approval
Amandine Martin	Acquisition, analysis of data + final Approval
Michel Popoff	Critical material for the study + final approval
Mathieu Gerfaud-Valentin	Critical material for the study + clinical info + final approval
Pascal Sève	Critical material for the study + clinical info + final approval
Alexandre Belot	Critical material for the study + clinical info + final approval
Anne Contis	Critical material for the study + clinical info + final approval
Agnes Duquesne	Critical material for the study + clinical info + final approval
Gaetane Nocturne	Critical material for the study + clinical info + final approval
Irene Lemelle	Critical material for the study + clinical info + final approval

DECLARATION OF CONTRIBUTIONS TO ARTICLE

ADMC

Manuscript Number: [ ]

Journal Name: *Cell Death & Differentiation* (the 'Journal')

Proposed Title of the Contribution: [ ] (the 'Contribution)

Author(s): [ ] (the 'Authors')

For all *CDD* articles, each person named as an author in the published version must be able to show he or she has contributed substantially to the article.

Authorship credit should be based on 1) substantial contributions to conception and design, acquisition of data, or analysis and interpretation of data; 2) drafting the article or revising it critically for important intellectual content; and 3) final approval of the version to be published. Authors should meet conditions 1, 2 and 3.

Any person who cannot be shown to have made a substantial contribution to the article cannot be listed as an author in the final version. The name of any person who is deemed to have made a minor contribution can, however, appear in the Acknowledgments section of the article.

Please complete the table below to indicate the contributions of all named authors to the manuscript.

Author Full Name:	Specification of Contribution to the Manuscript:

Please complete the table below to indicate the contributions of all named authors to the figures.

Figure 1:

A: DC
B: DC, MP
C: DC, MP
D: DC
E: DC
Supervision YJ-TH

DC performed expts A-E; MP produced TcdA, DC analyzed and assembled the figures

Figure 2:

A:DC
B:DC
C:DC, AM
D: DC, AM
E: DC, AM
F:DC
Supervision YJ-TH

DC performed expts A-B, F; AM performed expts C-E; DC analyzed and assembled the figures

Figure 3:

A: DC
B: DC, AM
C: DC, FM, AM
D: DC, FM, AM
Supervision YJ-TH

DC performed expt A, AM performed exptsB-D, FM performed expt C-D; DC analyzed and assembled the figures

Figure 4:

A: DC
B: DC, MP
C: DC, MP
D: DC, MP
E: DC, PB
F: DC, PB
G:DC
H: DC, PB
I: DC, PB
J: DC, PB
Supervision YJ-TH

DC performed expt A-D, G, PB performed expts E-F, H-J, MP produced TcdA; DC analyzed and assembled the figures

Figure 5:

A: DC, SD
B: DC, SD
C:DC
Supervision YJ-TH

SD performed expt A-B, DC performed expts C; DC analyzed and assembled the figures

Figure 6:

A-J: FM, DC, MGV, PS, AB, AC, AD, GN, IL, SGL, GB, IT
Supervision YJ-TH

FM performed expt A-J with the help of DC,
GB and IT analysed database, identified patients and MDs, analysed genetic info
MGC, PS, AB, AC, AD, GN, IL, SGL, YJ recruited patients, provided primary cells, analysed clinical info

Signed for and on behalf of the Author(s):	Print Name:	Date:
	HENRY	2022-10-25
